# Recent advances in ultrafast plasmonics: from strong field physics to ultraprecision spectroscopy

**DOI:** 10.1515/nanoph-2021-0694

**Published:** 2022-03-21

**Authors:** San Kim, Tae-In Jeong, Jongkyoon Park, Marcelo F. Ciappina, Seungchul Kim

**Affiliations:** Department of Cogno-Mechatronics Engineering, College of Nanoscience and Nanotechnology, Pusan National University, 2 Busandaehak-ro 63beon-gil, Busan 46241, South Korea; Engineering Research Center for Color-modulated Extra-sensory Perception Technology, 2 Busandaehak-ro 63beon-gil, Busan 46241, South Korea; Physics Program, Guangdong Technion – Israel Institute of Technology, 241 Daxue Road, Shantou, 515063, Guangdong, China; Technion –Israel Institute of Technology, Haifa, 32000, Israel; Institute of Physics of the ASCR, ELI-Beamlines Project, Na Slovance 2, 182 21 Prague, Czech Republic; Department of Optics and Mechatronics Engineering, College of Nanoscience and Nanotechnology, Pusan National University, 2 Busandaehak-ro 63beon-gil, Busan 46241, South Korea

**Keywords:** optical frequency comb, photoelectron spectroscopy, strong-field physics, surface plasmons, ultrafast plasmonics

## Abstract

Surface plasmons, the collective oscillation of electrons, enable the manipulation of optical fields with unprecedented spatial and time resolutions. They are the workhorse of a large set of applications, such as chemical/biological sensors or Raman scattering spectroscopy, to name only a few. In particular, the ultrafast optical response configures one of the most fundamental characteristics of surface plasmons. Thus, the rich physics about photon–electron interactions could be retrieved and studied in detail. The associated plasmon-enhanced electric fields, generated by focusing the surface plasmons far beyond the diffraction limit, allow reaching the strong field regime with relatively low input laser intensities. This is in clear contrast to conventional optical methods, where their intrinsic limitations demand the use of large and costly laser amplifiers, to attain high electric fields, able to manipulate the electron dynamics in the non-linear regime. Moreover, the coherent plasmonic field excited by the optical field inherits an ultrahigh precision that could be properly exploited in, for instance, ultraprecision spectroscopy. In this review, we summarize the research achievements and developments in ultrafast plasmonics over the last decade. We particularly emphasize the strong-field physics aspects and the ultraprecision spectroscopy using optical frequency combs.

## 1 Introduction

Surface plasmons (SPs) are collective oscillations of electrons at conduction bands that react strongly when driven at their resonance wavelength. The latter is determined by the geometrical and physical parameters of both the metal and surrounding dielectric materials [1, 2]. A strong plasmonic interaction can significantly enhance the optical power at a spatially localized area, under such mentioned resonant conditions. Modern fabrication methods, with sophisticated process technology, allow such well-tailored plasmonic structures that optimally satisfy the surface plasmonic resonance (SPR) conditions. Furthermore, the plasmonic resonance is sensitive to the change in the environmental dielectric constant, exploited in refractive-index sensors, surface-enhanced Raman-scattering spectroscopy (SERS), and high-sensitivity molecular detection [3], [4], [5]. Therefore, the fundamental concepts of SPs have been applied across all fields including biology [6, 7], chemistry [8], [9], [10], [11], and physics [12], [13], [14]. We refer SPs typically as plasmonics, due to their special characteristics, such as the plasmonic field enhancement under resonance conditions. Plasmonics has been established as the core of various nanoscience and nanotechnology fields, including SPR sensors [15], strong field nano-optics [16], novel optical devices [17, 18] and metasurfaces [19, 20], and nanolithography [21].

Another remarkable field in modern physics is the so-called ultrafast optics. It investigates the physical or chemical phenomena at ultrashort time scales (fs or sub-fs) using femtosecond laser pulses. In general, a femtosecond pulse is obtained using mode-locking techniques to induce a fixed phase condition between the longitudinal optical modes in the laser’s resonant cavity. Various mode-locking techniques allow a multimode operation, where the phase is locked, resulting in a train of ultrashort pulses in the time domain. The optical spectrum of a pulse train consists of a series of equally-spaced, discrete optical frequency components in the frequency domain, which equals the pulse repetition rate, i.e. it configures a so-called optical frequency comb. Ultrashort pulses permit the observation of chemical reactions and electron dynamics in atoms, molecules, and, recently solids, in real time. Such optical frequency combs, referenced to the radio frequency (RF) of an atomic clock, enable precise measurement of optical frequencies, allowing ultraprecision molecular spectroscopy or nanoscale absolute distance measurements.

Ultrafast plasmonics, an exciting and novel research area in plasmonics, is the rendezvous of ultrafast optics and plasmonics. It has been a rapidly explored research area, started by studying the interaction between femtosecond electromagnetic (EM) fields and solid-state nanostructures. Ultrafast plasmonics pays attention to the behavior of the electron motion affected by the dynamical intensity and phase of the plasmonic field rather than monitoring the static SP-induced physical effects.

Ultrafast plasmonics has been extensively applied in modern physics (nanophotonics and nonlinear optics). For instance, plasmonics have been recently extended to the strong-field physics regime. Thus, it is now possible to investigate the underlying physics in various targets (atoms, molecules, and solids) under the action of intense EM fields without the need for expensive and big laser amplifiers. Strong-field physics phenomena cannot be explained by invoking perturbative theories. This is because the associated EM fields’ strengths of the driving sources attain values comparable to the ones that bind electrons inside atoms and molecules. One of the most prominent examples is the high-order harmonic generation (HHG). Here, a frequency comb, with odd multiple frequencies of the driven laser, is generated when a relatively high intense laser field ( $∼10^{13}-10^{14}$ W/cm^2^) is focused into a target (atom, molecule, or solid). Using plasmonics, one could take advantage, in principle, of the laser field enhancement due to the strong spatial confinement and observe HHG at a much lower laser intensity regime ( $∼10^{10}-10^{11}$ W/cm^2^). There are, however, several issues to be solved. For instance, a significant part of the optical energy in metals is dissipated as heat, related to ohmic losses, but its time scale is rather slower than the ultrashort driven pulse, permitting an instantaneously strong plasmonic field amplification.

Nowadays, ultrafast plasmonics has reached great maturity and has been extended to various research branches. Thus, all the achieved new properties and advantages of ultrafast optics and plasmonics cannot be compressed in a single article. Therefore, this review only presents specific examples of ultrafast plasmonics in strong-field physics [12, 13] and ultraprecision spectroscopy [14]. These applications have significantly contributed to the development of each research field and presented a new vision through a fusion of advantages: the ones of ultrafast optics, such as ultrashort pulses, sufficient peak power in the time domain, precision, stability, and broad spectral bandwidth in the frequency domain, and those of plasmonics, such as the strong field enhancement and high physical/chemical sensitivity of the plasmonic optical fields when disturbed by the environment.

We describe the development trends as well as the fundamental physics of ultrafast plasmonics in time and frequency. We hope this review will provide helpful concepts and technical approaches for the further development and new applications of ultrafast plasmonics.

### 1.1 Plasmonics

The SP, the excitation at the interface between two materials (metal and dielectric), where the real part of the dielectric function has the opposite sign, is a very well-known phenomenon. The physical properties of SPs depend on the geometry of the nanostructure (shape, size, and sharpness) and the material’s composition (dielectric functions at a given wavelength). Various SP modes, such as the plasmonic gap mode [22, 23], surface lattice plasmon [24], [25], [26], Fano-resonance [27], [28], [29], [30], and whispering gallery modes (WGMs) [31, 32], were demonstrated through such structural/material design. These various SP modes enable unique plasmonic effects such as the manipulation of light and field amplification in the subdiffraction region [33], [34], [35], [36], [37], [38], [39].

**Figure 1:**
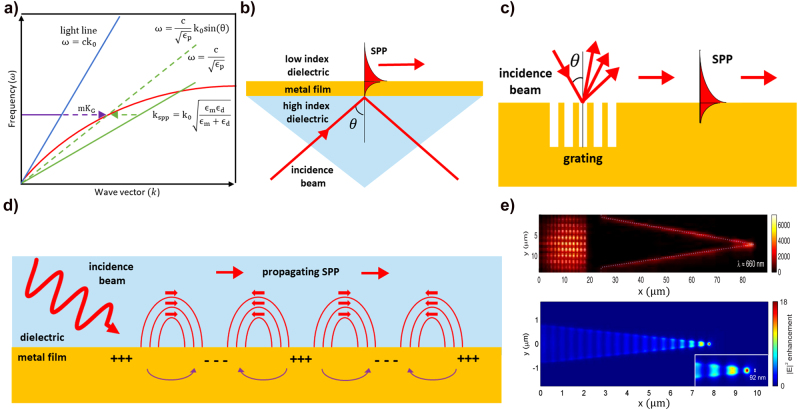
SPP excitation method and application. (a) Dispersion curve of the SPP and wave vector of the incidence beam at the dielectric/metal interface. SPP excitation method. (b) Kretschmann configuration, (c) grating coupling geometry. (d) Schematic of SPP propagating at the interface between the dielectric and the metal. (e) Nanoscale focusing of SPP by propagating through tapered structures. Upconversion luminescence image of SPP fields at the tapered plasmonic structure (upper panel), numerical simulation of focused SPP at V-groove structure (lower panel) Adapted with permission from ref. [49]. Copyright 2008, OSA.

Depending on the plasmonic formation, the SP can be classified into propagating surface plasmon polariton (SPP) and localized surface plasmon resonance (LSPR). The SPP propagates through the dielectric/metal interface by coupling with the induced EM waves. LSPR refers to a confined electric field around a nanostructure that couples the electrons in the metal and EM waves [40], [41], [42], [43]. The SPP requires a specific condition to be generated: the momentum of the incident beam and the SPP should match. The dispersion relationship of the SPP to fit the momentum with the incident EM wave can be derived by calculating the transverse magnetic EM wave in the dielectric/metal boundary condition using Maxwell’s equations. The SPP dispersion relation can then be expressed as

(1) $$k_{\text{spp}}=k_{0}\sqrt{\frac{\varepsilon_{\text{m}}\varepsilon_{\text{d}}}{\varepsilon_{\text{m}}+\varepsilon_{\text{d}}}},$$

where $k_{\text{spp}}$ , $k_{0}$ , $\varepsilon_{\text{m}}$ , and $\varepsilon_{\text{d}}$ represent the mode propagation constant, the light wavevector, the relative permittivity of the metal, and the relative permittivity of the dielectric, respectively. Figure 1a shows the wavelength-dependent dispersion plot of the common SPP propagation constant and wave vector of the incident light at the dielectric/metal interface. The calculation shows the SPP dispersion curve (red line) on the right of the respective light lines (blue line) of dielectric materials (e.g., air or SiO_2_). Thus, special techniques are required to satisfy the phase-matching conditions, such as prism coupling or grating for their excitation. Figure 1b shows the representative prism-based phase matching method, i.e., the Kretschmann configuration. This is a three-layer system comprising a thin metal film between two insulators of different dielectric constants. An incident beam reflected at the interface between the high dielectric constant medium (prism) and metal has an in-plane momentum adequate to excite SPP at the interface between the metal and the lower-index dielectric (green line). The horizontal wave vector component $\left(k_{x}=k_{0}\sqrt{\varepsilon }\;\sin\;\theta \right)$ at the prism/metal interface is matched with the propagation constant *k*_spp_ by adjusting the angle (θ) of the incident beam (green dash line). When the SPP phase-matching condition is satisfied, the evanescent excitation of SPP takes place at the thin metal film with an attenuated reflection of the incident beam.

The mismatch of the wave vector between the SPP and the incident beam can be resolved by fabricating the metal surface with equally spaced grooves. Figure 1c shows a grating coupling geometry for SPP excitation. Grooves provide additional momentum (purple dot line) to the incident beam momentum (purple line). Thus, the phase-matching condition using grating is expressed as

(2) $$k_{\text{spp}}=k_{0}\;\text{sin}\;\theta +mk_{\text{G}},$$

where $k_{\text{G}}=2\pi /a$ , with *a* the periodicity of grating, represents the additional grating momentum, with *m* an integer. The phase-matching condition is satisfied by adjusting the incidence angle θ . For details on the phase-matching scheme and mathematical analysis, we recommend referring to these review papers [44, 45].

The strength of the SPP field exponentially decays with an increase of the distance from the interface, and it propagates along with the interface between both media confined in a very narrow region (Figure 1d). This distinctive propagating feature of SPP allows focusing and manipulating optical energy at the nanoscale beyond the diffraction limit; this was difficult to achieve in a bulk optical system [46], [47], [48]. Figure 1e shows an example of the experimental (upper) and numerical simulation (down) of the nanoscale focusing on tapered geometries [49]. The concentration of SPPs is possible by shaping the three-dimensional geometry of the interface because propagating SPPs are bounded at the interface. In this scheme, it was demonstrated the excitation of SPP by grating coupling and the nanofocusing of SPPs at the apex of the tapered geometry. Various studies (including the nanoscale focusing of SPP) utilizing SPP are underway; for example, SPP waveguide designs (grooved structures [50, 51], gratings [52, 53], and bilayers [54, 55]), miniature optoelectronic devices [56, 57], and photonic circuits [58], [59], [60].

Another exciting application of SPPs is the extraordinary optical transmission (EOT) in subwavelength holes milled on a metal film; the magnitude of EOT here is several orders more than Bethe’s prediction, where the transmission through a subwavelength circular hole scales proportionally to (*r*/*λ*)^4^, where *r* denotes the hole radius and *λ* the driving wavelength [61], [62], [63]. The additional momentum of the periodic hole array induces the coupling of SP and light, and the resultant EM waves resonate on the surface; this increases the optical transmission through the film. EOT-based nanohole arrays have been used in various fields such as chemical/biological sensors [64, 65], SP-active devices for optoelectronics [66, 67], and metamaterials [68].

**Figure 2:**
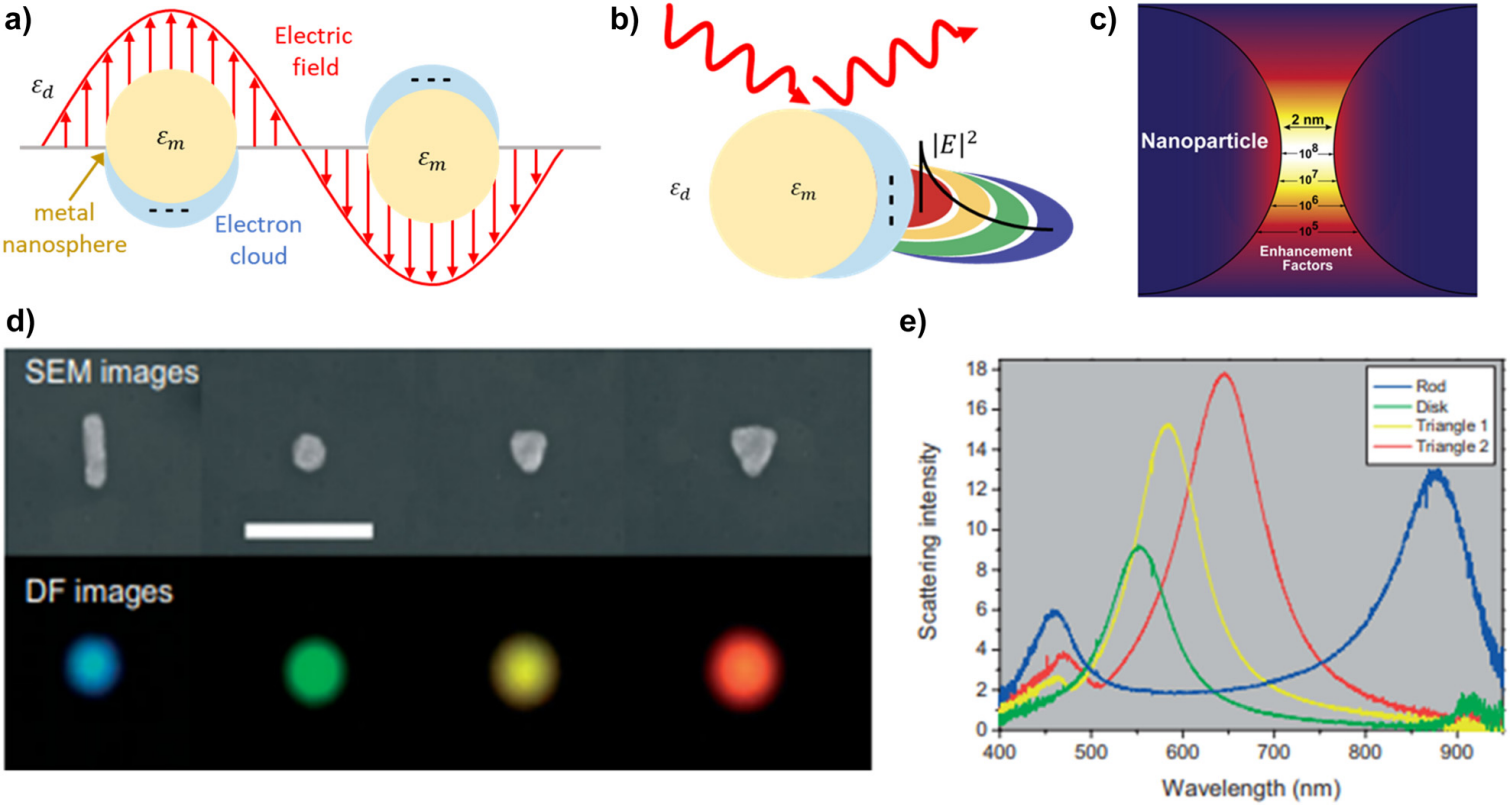
The formation process and characteristics of LSPR. (a) Formation of LSPR in metallic nanospheres. LSPR is effectively generated when the conduction band electrons in the metal nanosphere oscillate in phase with the incident electric field. (b) Schematic of the electric field distribution near the metal nanosphere; the LSPR induced electric field exponentially decays from the surface of the nanosphere. (c) Schematic of the hot spot between nanoparticle dimers showing the dramatically enhanced local EM fields. Adapted with permission from ref. [72]. Copyright 2011, Elsevier. (d) Several geometrical types of metallic nanoparticles, upper panel: SEM images, lower panel: dark field (DF) images, (e) scattering spectrum of each nanoparticle [42].

Unlike SPP, LSPR is the “non-propagating” resonance oscillation of the conduction electrons of metallic nanoparticles induced by external EM fields. Figure 2a shows the formation process of LSPR in nanoparticles. These electrons are steered by an external EM field and they gain an effective restoring force based on the shape of the nanostructure [69], [70], [71]. The two forces are resonant, which leads to a field amplification at the nanoparticle surface.

For the quantitative analysis of LSPR, a quasi-static approximation is adopted in that the nanoparticle size is considerably smaller than the wavelength of the light. In this case, the dipole moment can be used to compute the plasmonic field strength around the nanoparticle. The dipole moment *p* in the nanoparticle under the external EM field is expressed as

(3) $$p=\varepsilon_{0}\varepsilon_{\text{d}}\alpha E_{0},$$

where $\varepsilon_{\text{d}}$ , α , and $E_{0}$ represent the dielectric constant of the surrounding medium of the nanostructure, the polarizability, and the incident electric field peak strength, respectively. The polarizability α can be expressed differently depending on the nanoparticle geometry. In the quasistatic approximation, the polarizability of a nanosphere can be expressed as

(4) $$\alpha =4\pi r^{3}\frac{\varepsilon_{\text{m}}-\varepsilon_{\text{d}}}{\varepsilon_{\text{m}}+2\varepsilon_{\text{d}}},$$

where *r*, $\varepsilon_{\text{m}}$ , and $\varepsilon_{\text{d}}$ represent the particle radius, the dielectric constant of the nanosphere, and the dielectric constant of the surrounding medium, respectively. Theoretically, this equation indicates that a maximum in the polarizability occurs at $\varepsilon_{\text{m}}+2\varepsilon_{\text{d}}=0,$ when the imaginary part of the dielectric constant is ignored. This implies distinct LSPR features appear around the wavelength satisfying such a condition. In general, the real part of a dielectric constant is utilized to estimate the resonance peak wavelength of LSPR, while the imaginary part of the dielectric function is related to the damping and resonance peak broadening. For detailed mathematical descriptions, we recommend referring to [40, 72]. The intensity distribution of LSPR is similar to the case of SPP in that the electric fields exponentially decay from the surface of the nanoparticle, as indicated in Figure 2b. However, the field strength and distribution of LSPR can be further enhanced by positioning another nanoparticle closely, which induces a plasmonic coupling. Figure 2c shows the electrical hot spot from the localized plasmonic coupling effect in the overlapped decay region. The electric field of the hot spot increases with a decrease in the distance between the nanostructures [73, 74]. Figure 2d and e shows the SEM/dark-field images and the scattering spectrum from the rod, circle, and triangle with different sizes, respectively. The resonance wavelength of the nanoparticles is greatly affected by not only their size but also their shape [75], [76], [77].

### 1.2 Ultrafast pulsed lasers

An ultrafast pulsed laser emits ultrashort pulses on the order of femtoseconds (1 fs =10^−15^ s). These pulses are operated by a mode-locking technique [78, 79], that induces individual longitudinal modes in the laser cavity to be phase-locked through various methods. The mode-locking technique is classified into active and passive, depending on the phase control method [80]. Active mode-locking is conducted by inserting an electro-optic modulator (EOM) or acousto-optic modulator (AOM) in the laser cavity. The EOM-based mode-locking modulates the field amplitude temporally, like a periodic shutter, with the cavity round trip time. This modulation frequency (time) corresponds to a longitudinal mode spacing. The AOM-based mode-locking is performed by injecting adjunct longitudinal modes in the laser cavity, that is frequency shifted acousto-optically. The frequency-shifted mode spacing corresponds to the laser cavity mode spacing, *c*/*2L*, where *c* is the speed of light and *L* is the length of the cavity. Each injected mode has a common phase relationship with the existing modes in the cavity in such a way that a stable pulse laser operation is achievable.

Passive mode-locking techniques are conducted by saturated absorbers or Kerr media. For passive mode-locking using a saturable absorber medium, different optical transmissions, depending on the incident light intensity, are allowed. Low-intensity light has a low transmittance because of being absorbed through the medium; however, a sufficiently high-intensity light can pass through the medium with high transmittance. When light with sufficient intensity is formed in the laser cavity by passively controlling the cavity-losses through this optical property of the saturable absorber, the cavity-losses are rapidly lowered, and the intracavity modes, whose phases are fixed, are generated instantly from the medium. All of these emitted in-phase modes generate an ultrashort pulse.

A Kerr medium is placed in the laser cavity to induce a self-focusing effect according to the refractive index change by the nonlinear Kerr effect. For passive mode-locking using a Kerr medium with a high *q*-factor, the Kerr effect is expressed as

(5) $$n=n_{0}+n_{2}I,$$

where *n*, *n*_0_, *n*_2_, and I denote the changed, fundamental, second nonlinear refractive index, and light intensity, respectively. In general, most laser sources have a Gaussian beam profile whose form is such that the light intensity decreases from the center to the edge of the beam. Therefore, a sufficient high-intensity light can considerably change the refractive index based on the spatial distribution of the beam intensity when it passes through the Kerr medium; this provides not only a self-focusing effect called the Kerr lens in space but also a self-phase modulation by group delay dispersion (GDD) in the time domain. Finally, a diaphragm, through which only the focusing beam can pass, is placed to spatially block the continuous-wave (CW) light irrelevant to the formation of ultrashort pulses; only pulses that have passed through the diaphragm can be amplified and emitted from the laser cavity.

When the phase correlation between intracavity modes is not fixed in the mode-unlocked conditions, the superimposed optical modes (each optical frequency) with random phases result in a laser emission with irregular intensity in time. For a mode-locked operation using the aforementioned methods, all intracavity modes can have a fixed phase relationship, thereby allowing the generation of a train of ultrashort pulses [79].

#### 1.2.1 Mode-locked femtosecond laser in the time domain

The pulse duration of a mode-locked laser pulse is determined by the number of in-phase optical frequency modes in the laser cavity; it can be expressed as [78]

(6) $$\Delta t=\frac{TBP}{N\Delta \nu },$$

where Δ*t*, *N*, ∆*ν*, and *TBP* denote the pulse duration, number of in-phase optical modes, frequency difference between nearest optical frequency modes, which is the reciprocal to the period, and the time-bandwidth product, respectively. The *TBP* is determined by the shape of the pulse envelope, and, for a representative Gaussian-shaped envelope, has a value of 0.441. Another pulse shape, hyperbolic–secant–squared, has a value of 0.315. We can estimate the pulse duration using Eq. (6), which has a value in the sub-picosecond range. The development of femtosecond pulsed lasers has been actively explored for several decades since the first proposal of the mode-locking technique in 1964 [81]. For a solid-state titanium-doped sapphire (Ti:sapphire) laser, which is the most general femtosecond pulsed laser, it is possible to emit ultrashort pulses with a pulse duration of ∼10 fs [82] and even shorter, ∼5 fs [83, 84], through an additional extreme optimization setup in/out of the cavity.

**Figure 3:**
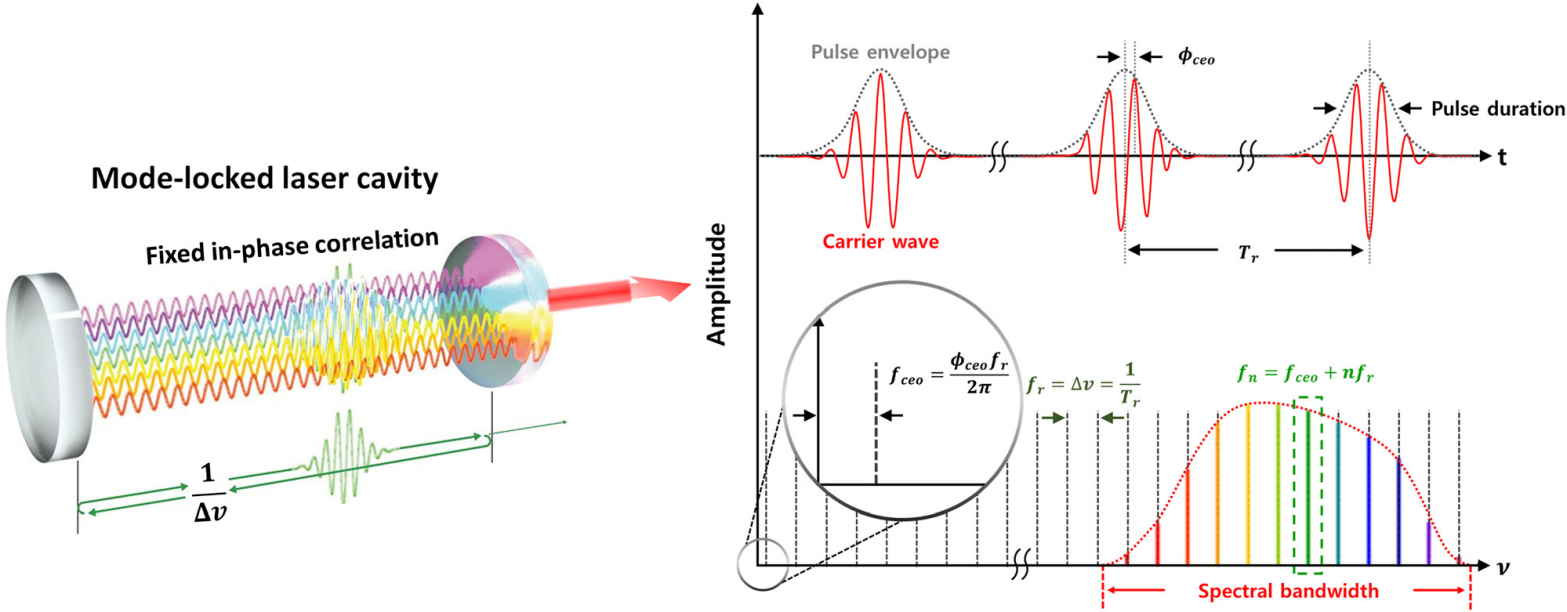
Characteristics of mode-locked ultrafast lasers in the time and frequency domains. Each intracavity mode is expressed as $f_{\text{n}}=f_{\text{ceo}}+nf_{\text{r}}$ in the frequency domain, which provides a broadband frequency comb while adding to form an ultrashort pulse train in the time domain with a period $T_{\text{r}}$ , which is the inverse of the repetition rate $f_{\text{r}}$ . The slightly different phases of each carrier wave generate the $f_{\text{ceo}}$ shown as $f_{\text{ceo}}=\frac{\phi_{\text{ceo}}f_{\text{r}}}{2\pi }$ [215].

The pulse duration according to Eq. (6) is closely related to the intracavity length and the number of in-phase modes. Further, the cavity length relates to the pulse repetition rate $f_{\text{r}}$ according to the formula $f_{\text{r}}=c/\left(2L\right)$ . Therefore, the intracavity optical frequency modes exist in the form of harmonics with the frequency spacing of Δ*ν*, and the bursting pulse trains with the repetition rate $f_{\text{r}}\left(1/T_{r}\right)$ , where $\Delta v=f_{\text{r}}$ , as shown in Figure 3. Most mode-locked femtosecond lasers have repetition rates ranging from the tens of megahertz (1 MHz = 10^6^ Hz) to several gigahertz (1 GHz = 10^9^ Hz) determined by the cavity length. The number of in-phase modes *N* can be considered the spectral bandwidth of the laser because intracavity modes within the gain bandwidth only survive by being pumped through the stimulated emission mechanism in the gain medium. Therefore, the number of in-phase modes can be derived from the spectral bandwidth of the laser and optical mode spacing. For the same average power of the laser, the energy of each pulse is determined based on the pulse repetition rate; the pulse energy/pulse duration provides the instantaneous peak power. Thus, based on the above properties, the mode-locked femtosecond laser is expressed in the form of a femtosecond-scaled pulse train at regular intervals.

For extreme nonlinear optical experiments like HHG, the femtosecond pulse energy needs to be strongly enhanced [85]. However, most pulsed lasers have a considerably stronger peak intensity than general CW lasers; therefore, optical elements in a pulsed laser system easily suffer thermal/nonthermal damage. The chirped pulse amplification (CPA) technique has been suggested as a breakthrough for amplifying a femtosecond pulse laser while avoiding the optical damage induced by the peak intensity of the pulse laser [85, 86]. CPA is conducted as follows: the fundamental pulse is spectrally dispersed using a pair of gratings that disperses the spectrum and stretches the pulse in time (e.g. forming a ps or ns pulse) to prevent the laser-induced damage to the optical elements. Next, the energy of the dispersed pulse is amplified through an external gain medium. Finally, the amplified pulse is recompressed using a second pair of gratings, reversing the dispersion of the first pair while restoring a pulse duration in the femtosecond domain. Through these series of processes, ultrashort pulsed lasers with high peak intensities can be obtained.

#### 1.2.2 Time-domain measurements

**Figure 4:**
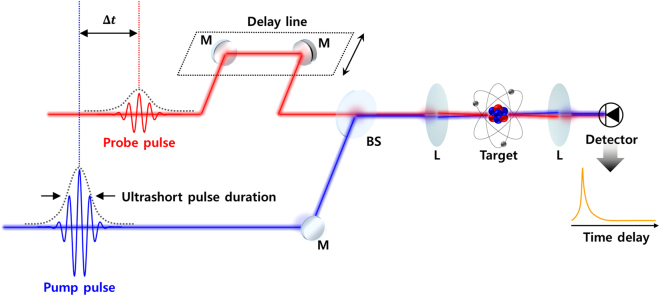
Typical experimental apparatus of an ultrafast pump-probe measurement. The pump and probe pulses, with ultrashort pulse duration, excite the target sample with a time delay. The time-resolved spectrum is obtained from an optical detector by scanning the delay line, which allows changing the time delay between the pump and the probe pulses. The pulse duration of the probe pulse limits the temporal resolution of the spectroscopy. M, mirror; BS, beam splitter; L, lens.

Most of the ultrafast time-domain measurements are based on the pump-probe technique as shown in Figure 4 [80]; it is a time-resolved measurement that obtains information on ultrafast phenomena generated in a sample using common picosecond or femtosecond-scaled ultrashort pulses. The general mechanism of the measurement is as follows [87]: an investigated sample is promoted to an excited state by an incident pump-pulse. Then, a probe pulse with a power lower than the pump is irradiated onto the sample. The time-resolved resultants upon the time difference between the pump and probe pulses can be achieved via measuring the reflected or transmitted probe pulse’s intensity or its spectral changes by scanning the delay line. This enables the observation of atomic and molecular-scaled damping dynamic processes at a femtosecond timescale by shifting the delay line (the time resolution results dividing the delay shift, typically several hundreds of nanometers, by the speed of light) [80].

Therefore, the pump-probe technique, typically not limited by the detectors’ response time, is suitable for observing ultrafast phenomena that are difficult to scrutinize using other approaches. However, the ultrafast pump-probe measurement should use a light source with an ultrashort pulse duration that does not degrade the femtosecond/attosecond-scaled delay scanning resolution. Various efforts have been invested in reducing the pulse duration from the femtosecond to the attosecond scale to monitor sub-femtosecond-scaled ultrafast phenomena such as the electron dynamics inside atoms and molecules [88, 89].

Attosecond pulses are obtained through the HHG process by focusing an intense femtosecond laser on a target material [90]. HHG is a coherent frequency upconversion phenomenon that allows the generation of ultrashort pulses at the extreme ultraviolet (XUV) range [91], [92], [93]. This nonlinear optical conversion process requires an intense field of light, which can be achieved by using the aforementioned CPA technique [94, 95] and other intracavity pulse amplification approaches [96], [97], [98]. However, CPA techniques experience a reduction of the repetition rate down to the several hundred kilohertz (1 kHz = 10^3^ Hz) level to minimize the thermal damage of optical elements.

Later, a strong field enhancement technique using SPs in ultrafast plasmonics was proposed; it is the non-CPA-based HHG, where a tailored plasmonic metal nanostructure provides a sufficient optical field enhancement. The strong field enhancement of the plasmonic effect amplifies the optical field up to several orders of magnitude [12], providing a sufficiently high intensity for HHG. Such a strong field generated through the combination of ultrafast optics and plasmonics allowed HHG without any other large amplification system, maintaining the original repetition rate, typically several tens of megahertz. Thus, plasmonic HHG is ideally suitable to not only observe sub-femtosecond scale ultrafast phenomena [99] but also develop high-resolution imaging techniques [100] and lithography applications [101].

#### 1.2.3 Mode-locked femtosecond laser in the frequency domain

Mode-locked femtosecond laser forms an optical frequency comb [102, 103] in the frequency domain that provides millions of well-defined comb lines with a high phase coherence in a broad spectral bandwidth, as shown in Figure 3. An optical frequency comb referenced to an atomic clock has been widely used as an ultraprecision optical source in optical metrology because of its high precision and stability [102], [103], [104]. The optical frequency comb is configured in a form where discrete comb lines are uniformly arranged with a certain spacing in the frequency domain; each comb line can be expressed as

(7) $$f_{\text{n}}=f_{\text{ceo}}+nf_{\text{r}},$$

where $f_{\text{n}}$ , $f_{\text{ceo}}$ , and $f_{\text{r}}$ denote the *n*th (*n* is an integer) comb line, carrier-envelope offset frequency, and repetition rate, respectively. As shown in Figure 3, $f_{\text{ceo}}$ is characterized in the time domain as a phase difference ∆*ϕ* between the pulse envelope position and carrier wave. Further, it can be generated by the GDD of the optical elements in the laser cavity. In the frequency domain, the phase difference ∆*ϕ* can be expressed as $f_{\text{ceo}}=\frac{f_{\text{r}}\Delta \phi }{2\pi }$ . The comb lines are positioned in a row with a spacing resulting from the repetition rate value over the spectral bandwidth of the gain medium; $f_{\text{ceo}}$ is the residual contribution of $f_{\text{r}}$ in each comb line which is graphically represented as the offset frequency, as shown in Figure 3. Therefore, $f_{\text{ceo}}$ can be between zero to values less than the repetition rate, $f_{\text{r}}$ . Therefore, each optical frequency comb can be defined as two RF frequencies to be easily linked to an atomic clock frequency.

**Figure 5:**
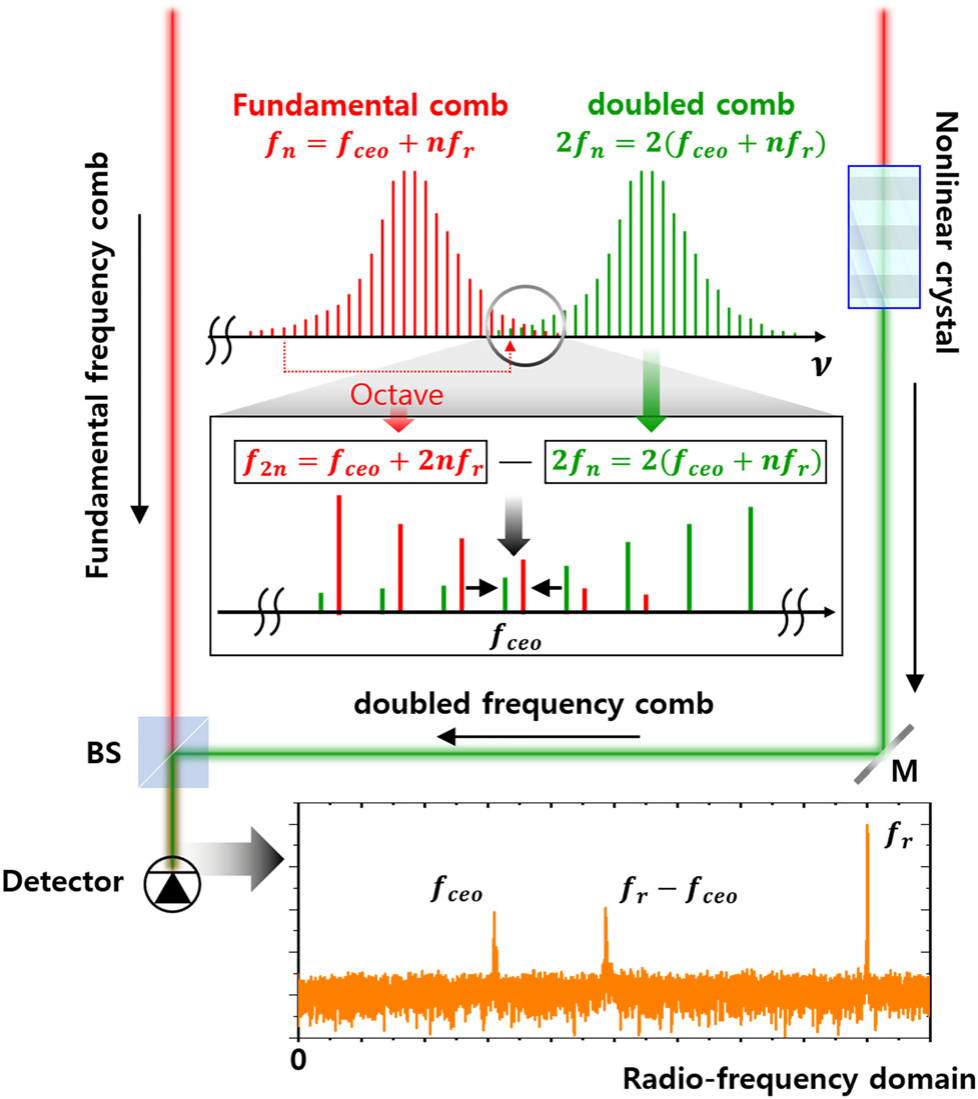
Working principle of the f–2f interferometric optical synthesizer. The *n*th mode of the optical frequency comb at the red line, whose frequency is expressed as $f_{\text{ceo}}+nf_{\text{r}}$ , is upconverted by a nonlinear crystal shown as the green line. The frequency of the upconverted comb is twice the frequency of the fundamental comb expressed as $2\left(f_{\text{ceo}}+nf_{\text{r}}\right)$ . The 2*n*th mode of the fundamental frequency comb $\left(f_{\text{ceo}}+2nf_{\text{r}}\right)$ interferes with the frequency-doubled comb mode $2\left(f_{\text{ceo}}+nf_{\text{r}}\right)$ when the optical frequency comb is assumed to covers a full optical octave. This yields the beat frequency, which is the carrier-envelope offset frequency $f_{\text{ceo}}$ in the RF domain.

Therefore, it is essential to stabilize $f_{\text{r}}$ and $f_{\text{ceo}}$ if one wants to use the frequency comb as a high precision light source [105]. $f_{\text{r}}$ is determined by the laser cavity length and can be precisely controlled by piezoelectric actuators. $f_{\text{ceo}}$ can be extracted through a well-known f–2f interferometer, as shown in Figure 5 [106]. The interferometric extraction process is described as follows: using a Mach–Zehnder interferometer, the optical frequency comb at one arm passes directly whereas the other one passes through a nonlinear optical medium such as a beta-barium-borate (BBO) crystal, inducing second-harmonic generation; i.e., the new frequency comb lines doubles the optical frequency comb. The optical interferometric beating between the original frequency comb in one arm and the frequency-doubled comb in the other arm generates an RF beat frequency, where their spectral regions are overlapped. This extracted RF beats using an f–2f interferometer correspond to the $f_{\text{ceo}}$ ; this frequency is encoded in the automatic feedback loop and controlled continuously through the optical elements of the laser cavity such as optical modulators and oscillators, which adjust the intracavity conditions based on the feedback values.

#### 1.2.4 Frequency-domain measurement

**Figure 6:**
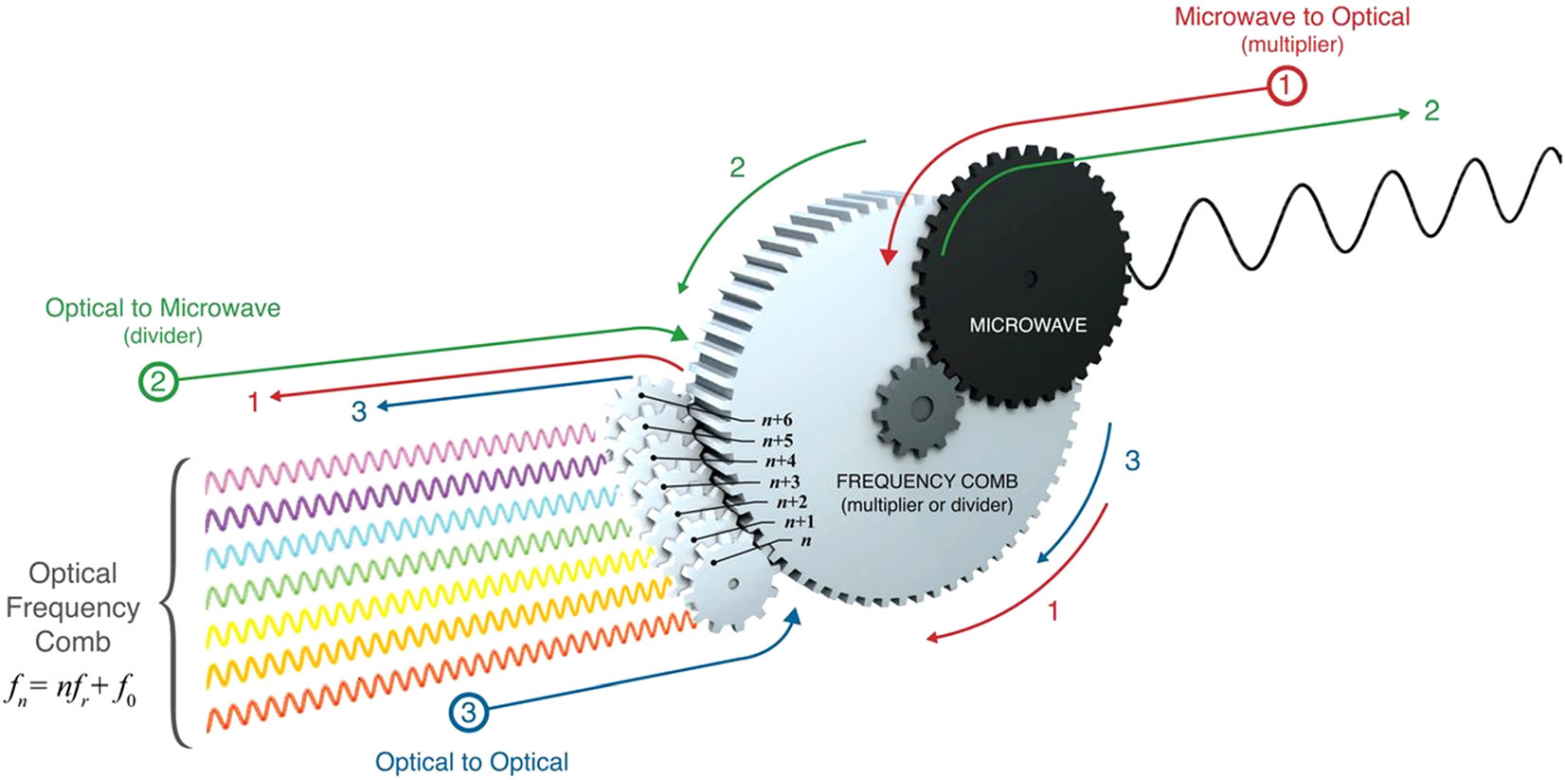
Metaphorical sketch of the correlation between optical and microwave frequencies by an optical frequency comb. The frequency comb provides the time standard while interconnecting the microwaves and optical frequencies coherently. The optical frequency comb has three bidirectional, simultaneous modalities, that can operate on phase coherently linking between the optical domain of the EM spectrum and the microwave domain of the electronics. As shown in red line “1,” the input microwave frequency is multiplied by about five orders of magnitude to appear in the optical frequency domain as equally spaced optical waves. The green line “2” shows the opposite mechanism to process “1,” and the input high-frequency optical wave is scaled down into the microwave region. Finally, blue line “3” describes the spectral transformation between an optical wave array. It occurs when an optical input field modulates the comb, and its phase is coherently coupled with the other frequencies of the optical field [215].

The frequency stabilization of a mode-locked femtosecond laser is expressed as an optical frequency reference composed of harmonic chains in the frequency domain [104]. Optical frequency combs are widely used in optical metrology such as in precise length metrology [107, 108], astronomical spectroscopy [109, 110], intercomparison of atomic clocks [111], and high resolution and precision spectroscopy [112], because of their properties such as high stability and precision which are attributed to being referenced to an atomic clock. This light source has numerous advantages such as broad spectral bandwidth and straightforward interconnection between optical and microwave frequencies as shown in Figure 6. The optical frequency comb has become a highly suitable light source for high precision spectroscopy. However, most of the basic spectroscopy measurements were performed by measuring the spectral intensity variation with diffraction gratings, which were affected inevitably by the geometric waveform distortion that occurs in experiments; this would make it difficult to fully exploit the benefit of an optical frequency comb for higher precision spectroscopy. Not like the colorimetric measurement, the phase measurement in an optical frequency for spectroscopy would be a considerably better choice to measure spectroscopic information in targets with an optical frequency comb; this is less affected by instrumental resolutions. The optical frequency is a high precision parameter because it is the reciprocal of time, and it can be measured considerably more accurately than any other physical quantity. Therefore, frequency analysis has been a key tool in spectroscopy, and frequency-based optical spectroscopy has been an active area of research [113, 114]. For frequency-based spectroscopy, an optical frequency comb has been found to be the optimal light source for ultraprecision spectroscopy because its frequency can be stabilized and manipulated precisely.

Over the past few decades, both the resolution and precision of spectroscopy have been significantly improved using an optical frequency comb; this has resulted in versatile applications in the areas of chemical and biological analysis [115]. However, researchers aim to further develop state-of-the-art technologies for identifying unknown processes in all scientific fields [116]. Recently, another version of ultraprecision spectroscopy using ultrafast plasmonics was demonstrated [14]. Ultrafast plasmonics can be advantageous for the next generation of spectroscopy techniques merging the properties, precision, and stability of the optical frequency comb with the high dynamical sensitivity of the plasmonic sample. Thus, the symbiosis of ultrafast optics and plasmonics enables the realization of ultraprecision spectroscopy beyond conventional methods.

The following section introduces the merged research that exploits the benefit of plasmonics in the time domain; the electric field amplification and its nanoscale spatial manipulation. The fundamental concepts, tied to the conventional strong-field physics, will be also discussed.

## 2 Ultrafast plasmonics and strong-field physics

Strong field physics studies the fundamentals of the phenomena that take place when atoms, molecules and solids are driven by an intense laser field. This research field focuses on probing and controlling the electron dynamics at atoms or molecules. A considerable amount of fundamental research has been conducted after the development of mode-locked lasers in the femtosecond regime. This tool is essential for the nonlinear light–matter interaction processes to happen. Later, strong field nano-optics have been proposed. Here, plasmonic effects, such as the enhancement or spatial manipulation of the laser electric field, are used to steer the electrons at the nanoscale.

### 2.1 Theory of high harmonic generation

**Figure 7:**
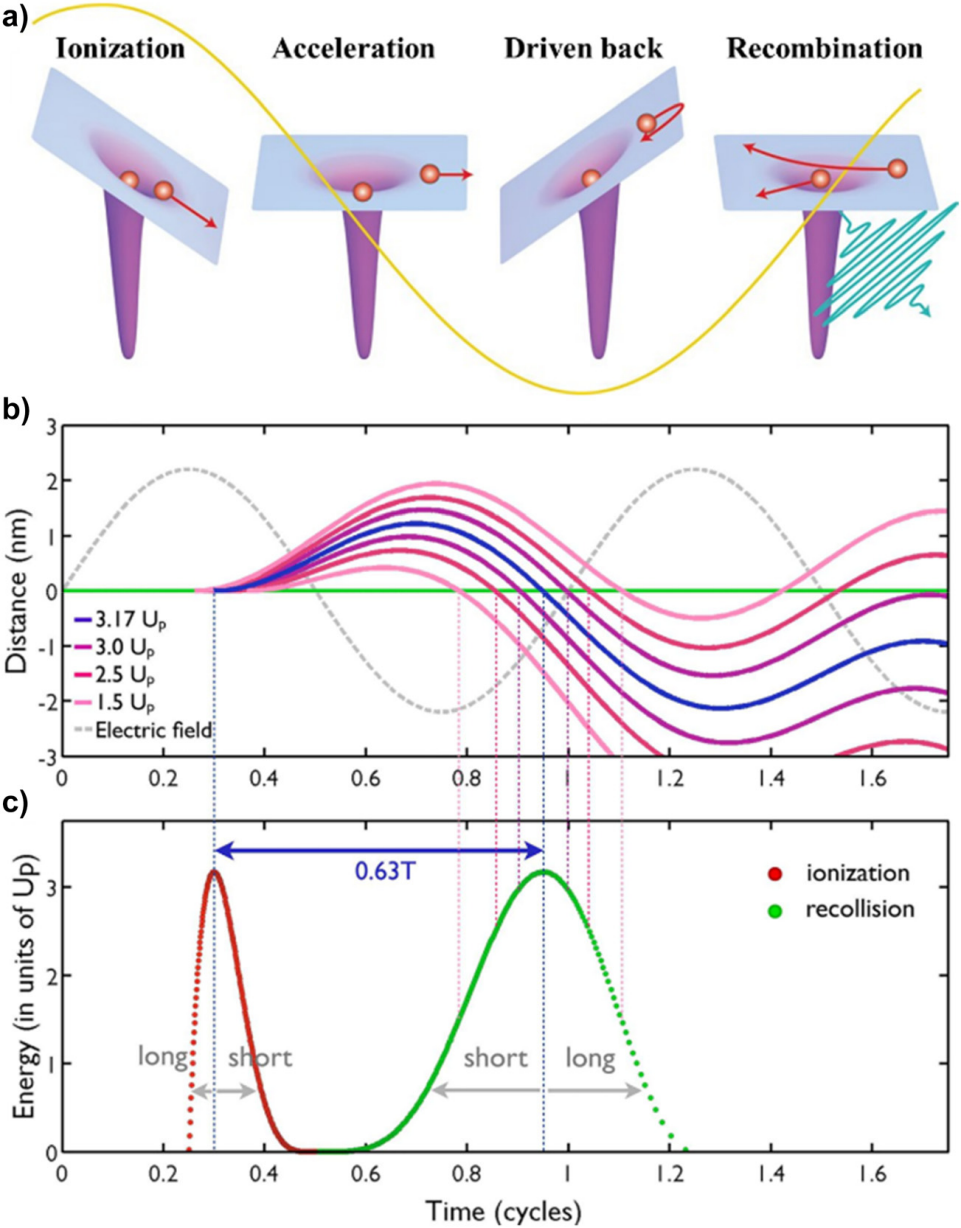
Three-step model and strong field approximation of HHG. (a) An illustration of the three-step model of HHG. The intense laser electric field distorts the potential barrier of an atom, which allows the tunneling ionization of electrons. The freed electrons are then accelerated by the laser electric field. They return close to the parent ion when the oscillating electric field reverses its direction, to finally recollide [119]. (b) Electric field-driven electron trajectory with different ionization times; the ionized electrons are accelerated and move away from the parent ion. The *y*-axis means the distance of the electron from the parent ion. When the direction of the electric field is reversed, the electrons return to the parent ion. (c) The resultant electron kinetic energy depends on the time of electron birth (red) and recollision (green) Adapted with permission from ref. [129]. Copyright 2013, Thesis of Salamanca.

When an intense laser field, with an associated electric field comparable to the one that binds the electron to the nucleus, is applied to an atomic target, tunneling ionization occurs. Here, the electrons pass through the potential barrier, formed by the atomic potential and the laser electric field, and escape from the atom. Tunneling ionization is the first step of various strong field-driven nonlinear processes, such as HHG. HHG is the workhorse of attosecond physics [16, 117, 118]. Around 30 years ago, Corkum suggested a three-step model, that simply explains the HHG process. Figure 7a illustrates the three-step model for HHG. The strong laser field momentarily modifies the potential of the atom, and the electrons are ionized by the tunneling effect [117]. The ionized electrons are then field-driven accelerated. Then, when the laser electric field reverses its direction, the electrons return close to the parent ion and recombine (recollide). Here, the energy gained during their journey in the laser-continuum is upconverted into high energy photons (HHG). If the electrons are elastically rescattered, they contribute to the high-order above-threshold ionization (HATI) process [117, 120], [121], [122], [123].

Typically, the ultrafast photoionization in atoms and molecules, the first step of the three-step sequence, is classified into multiphoton ionization and tunneling ionization, based on the electric field strength and the properties of the target material. The tunneling ionization mechanism has been experimentally proved, and it supports many strong field theories. Attosecond physics has opened up new research avenues, allowing researchers to study the electron dynamics in atoms, molecules and solids, with unprecedented temporal, attosecond, and spatial nanometric resolution.

#### 2.1.1 Keldysh parameter

According to the Keldysh theory, an electron can be freed from an atomic or molecular core either via tunnel or multiphoton ionization. These two regimes can occur depending on the medium and laser characteristics, i.e. the binding energy, laser intensity, and wavelength [117, 124], [125], [126]. The Keldysh parameter *γ*_k_ defines these regimes, and it can be expressed as

(8) $$\gamma_{\text{k}}=\sqrt{\frac{E_{\text{B}}}{2U_{\text{p}}}},$$

where *E*_B_ and *U*_p_ represent the binding energy of the atom and ponderomotive energy, respectively [127]. The ponderomotive energy refers to the mean kinetic energy of free electrons in an EM field; it can be written as

(9) $$U_{\text{p}}=\frac{e^{2}E_{0}^{2}}{4m_{\text{e}}\omega_{0}^{2}},$$

where e , $E_{0}$ , $m_{\text{e}}$ , and $\omega_{0}$ represent the electron charge, electric field amplitude, electron mass, and laser field frequency, respectively. For $\gamma_{\text{k}}$ > 1, multiphoton ionization occurs (perturbative regime, relatively low laser intensities) and the *N*th harmonic strength scales as the laser intensity to the power of *N* (*I* ^*N*^). In this perturbative regime, the *N*th harmonic intensity rapidly decreases with an increase in the harmonic order. On the contrary, tunneling ionization develops (non-perturbative regime, relatively high laser intensities) when $\gamma_{\text{k}}$ < 1. Here, plateau (the intensity of a set of *N*th harmonics remains relatively constant) and cutoff regions (there is no harmonic radiation beyond a certain *N*th harmonic) in the HHG spectrum appear.

#### 2.1.2 Strong field approximation

In the tunneling ionization process, the strong laser field is assumed to play a more dominant role than the atomic potential. Therefore, the strong-field approximation (SFA) ignores the atomic potential and analyzes the motion of electrons, once ionized, using solely the laser electric field [128]. Then, the motion of an electron in an oscillating laser field can be expressed as

(10) $$\frac{{\text{d}}^{2}x}{\text{d}t^{2}}=-\frac{eE_{0}}{m_{\text{e}}}\text{cos}\left(\omega_{0}t\right)\text{.}$$

The position of the electron *x*(*t*) and its velocity *v*(*t*) can be derived by solving Eq. (10) assuming the initial conditions *x*(0) = 0 and *v*(0) = 0, i.e. the electron is at the origin and starts its motion from rest. Figure 7b shows electron trajectories at different ionization times [129]. The grey-dashed line represents the electric field whereas the green line represents the nucleus position, located at the coordinate origin. It is striking to note that the maximum kinetic energy (or recollision energy) of the electron is 3.17 *U*_p_ with an excursion time of approximately 0.63 times (blue line) the period of the laser pulse (*T*). Interestingly, larger or shorter excursion times of the electron do not increase its kinetic energy. Figure 7c shows the calculated electron kinetic energy with different ionization and recollision times. The maximum kinetic energy at the recombination (3.17 *U*_p,_ when the accelerated electron recombines with the parent ion only at a certain time), determines the cut-off emission energy in HHG, $E_{\text{cut}-\text{off}}\left(\text{eV}\right)=I_{\text{p}}+3.17U_{\text{p}}$ [130], [131], [132]. The coherent light generated through the HHG process has a considerably shorter wavelength (higher photon energy) than a conventional femtosecond pulse laser. Therefore, such coherently phase-matched short-wavelength light is the so-called attosecond pulse, which configures nowadays a fundamental tool for attosecond science. Attosecond streaking measurements (a pump-probe technique using an attosecond pulse in the presence of an IR laser field) provide insight into the electron dynamics with unprecedent extremely short time resolution. The spectrum of HHG can be also used for probing the band structure of solid targets or revealing topological effects [133, 134].

### 2.2 SP-enhanced HHG

#### 2.2.1 SP-enhanced HHG in gas

The gas-phase-HHG process requires a laser intensity of at least $10^{13}-10^{14}$ W/cm^2^, which is not attainable by a moderate power femtosecond laser oscillator. Thus, the CPA method is mostly employed to achieve such high peak intensity with a microjoule level pulse energy [135]. However, the conventional CPA laser with ultrashort pulse duration has a low repetition rate (several hundred kHz) due to the thermal effects in the optical parts and efficiency of the pump laser. Ideally, an attosecond pulse, generated by HHG, with a high repetition rate would be a suitable probe pulse for most of the ultrafast pump-probe spectroscopy techniques. However, the reduction of the repetition rate caused by the CPA-laser-based HHG leads to weak signal counts for spectroscopy. At the same time, longer data acquisition times are required, to get a reasonable signal-to-noise ratio (SNR). These extensive measurement times indeed prohibit precise measurement in ultrafast spectroscopy. This is because undesirable systematic noises such as mechanical vibrations, electrical power fluctuations, or laser stability are unavoidable. In addition, a weak attosecond pulse, with a high repetition rate, is a promising source to avoid space-charge effects, undesired in attosecond photoelectron spectroscopy [136].

**Figure 8:**
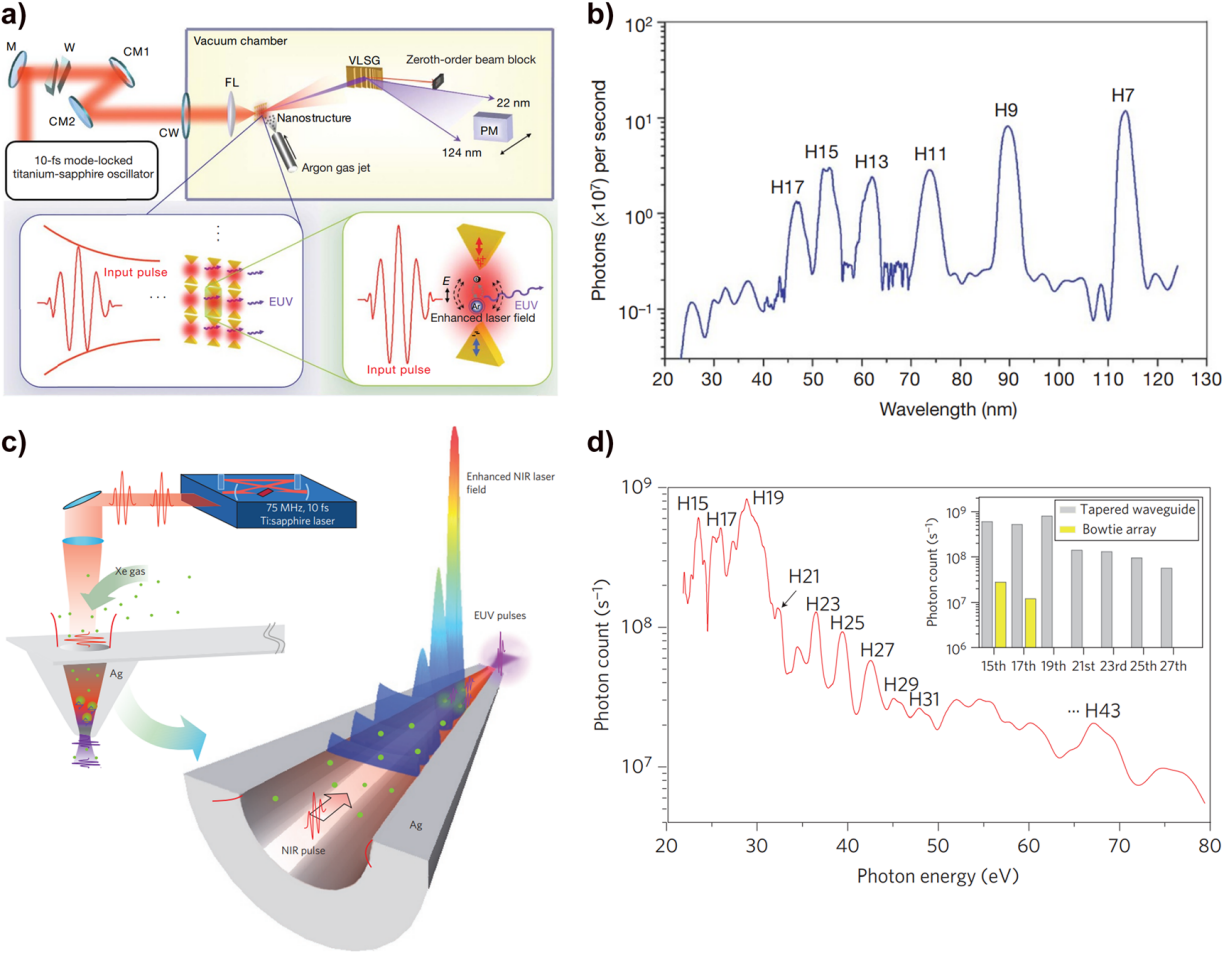
Plasmonically enhanced HHG in gases. (a) Schematic of experimental setup and mechanism of plasmonically enhanced HHG in an Au bowtie nanostructure array surrounded with Ar gas. The enhanced laser field by SPR from the bowtie nanostructure enables HHG. Ar, argon atom; CM, chirped mirror; CW, chamber window; FL, focusing lens; M, mirror; PM, photon multiplier; VLSG, varied-line-spacing grating; W, wedge plate; E, electric field [12]. (b) High harmonic spectrum from an Au bowtie nanostructure array surrounded with Ar gas. (c) Schematic of SP enhanced HHG in a 3D metallic waveguide filled with Xe gas. (d) High harmonic spectrum from the 3D metallic waveguide filled with Xe gas. The inset shows the comparison between the high harmonic photon yield of the 3D metallic waveguide filled with Xe gas (gray) and the Au bowtie structure array surrounded with Ar gas (yellow) [141].

In this respect, the plasmonic effect enables more than a hundred-fold field amplification, enough to induce HHG without the need of the CPA, permitting a MHz repetition rate XUV light source. Kim et al. experimentally proposed the SP-based HHG using a plasmonic nanostructure comprising an Au bowtie array to maintain the MHz repetition rate of the femtosecond laser oscillator [12]. Figure 8a shows the gas-phase HHG experimental setup and plasmonic field amplification process. The plasmonic enhancement can be induced at the gap of each bowtie by the interaction of the femtosecond pulse and the Au bowtie structure; this results in a maximum (numerically calculated) intensity enhancement of $2.5\times 10^{4}$ . Therefore, a focused Ti:sapphire laser with moderate output power and high repetition rate (10 fs, 800 nm, 75 MHz, $∼0.1\times 10^{12}$ W/cm^2^) reaches the intensity level to induce HHG in the injected Ar gas on the bowtie array. Generated high harmonic waves were dispersed by a varied-line-spacing grating, and the dispersed harmonic signal was recorded by scanning a photon multiplier laterally. Figure 8b shows the measured high harmonic spectrum. A clear cut-off at the 17th harmonics can be observed. The driving laser intensity $\left(∼0.1\times 10^{12}\;\text{W}/{\text{cm}}^{2}\right)$ is two orders of magnitude lower than the required intensity for gas-phase HHG; however, the induced SP in the bowtie nanoarray substantially amplifies the input intensity and allows gas-phase HHG to develop.

Later, Park et al. reported another SP-enhanced HHG setup, using a 3D metallic waveguide [141]. Figure 8c illustrates the concentrated SPP-based HHG mechanism and experimental setup. The 3D metallic waveguide has a hollow geometry to inject Xe gas atoms, which are the high harmonic emitters. The researchers exploited the SPP wave in their experiment, not the localized plasmonic resonance. The SPP waves generated at the interface between Ag and air propagate along with the tapered structure and concentrate on the tip when the near-infrared (NIR) beam is incident to the waveguide. A maximum intensity enhancement of $3.5\times 10^{2}$ , at the tapered tip, was numerically calculated. A Ti:sapphire laser pulse (75 MHz, 800 nm, $0.5\times 10^{12}$ W/cm^2^) was focused on the aperture of the tapered funnel structure, and Xe gas was injected to induce HHG. Figure 8d shows the measured high harmonic spectrum, having a cut-off at the 43rd order. The inset of Figure 8d shows a comparison of the high harmonic cut-off and photon count yield between the bowtie- and 3D-waveguide-based HHG. As can be observed, the 3D waveguide setup has a higher conversion efficiency and cut-off than the bowtie array one.

#### 2.2.2 Theoretical aspect of SP-enhanced HHG and limitations

**Figure 9:**
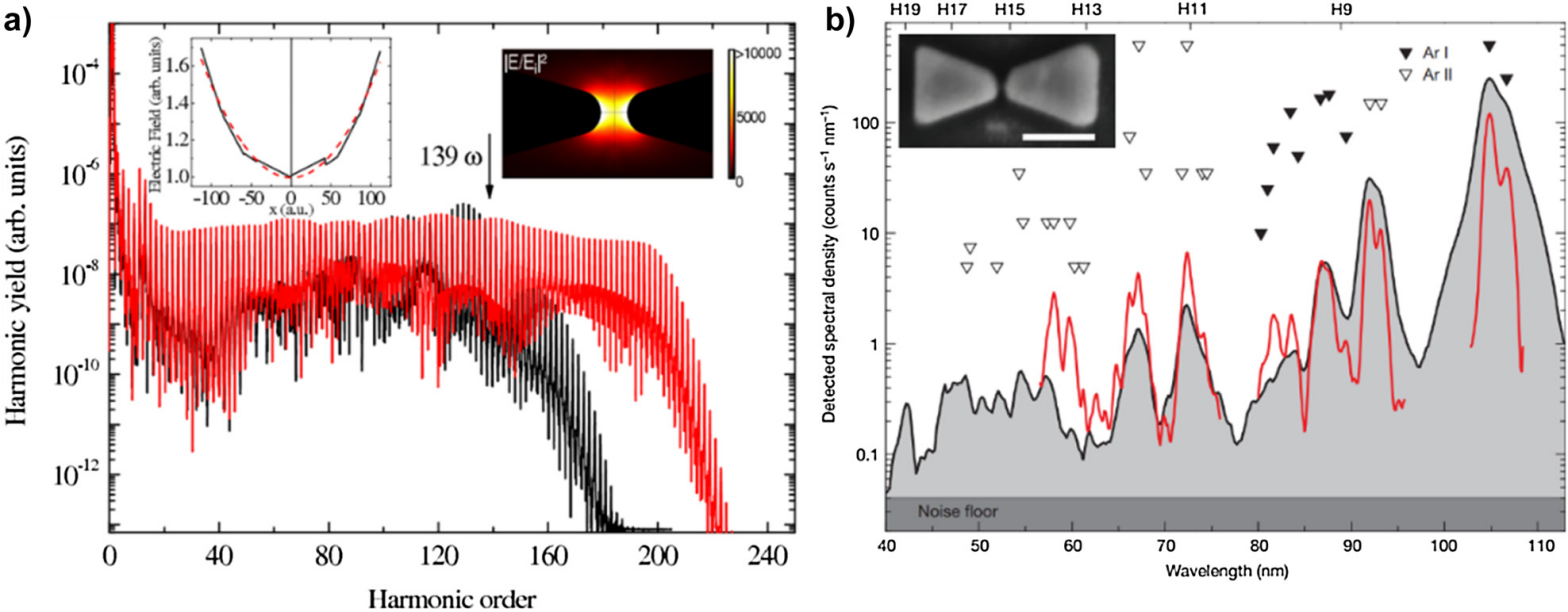
Numerical study and limitation of SP enhanced HHG in a gas. (a) Numerical simulation of the HHG spectrum from an Ar gas atom located at the center of the bowtie nanostructure calculated via the 1D TDSE, (ionization potential ℰ_GS_ = −15.7596 eV), at a peak intensity of *I* = 80 TW/cm^2^ and a bowtie gap of 12 nm. High harmonic spectrum with a homogeneous electric field (black) and a plasmonic-enhanced spatially inhomogeneous electric field (red). Calculated electric field distribution, *E*(*x*, *t*), at the bowtie gap (left inset), and intensity enhancement distribution at the bowtie structure (right inset) Adapted with permission from ref. [143]. Copyright 2012, OSA. (b) Atomic line emission spectrum from an Au bowtie structure array surrounded with Ar gas. The diffraction grating measured spectral density from the first diffraction order (black line) and second diffraction order (red line). The Ar atomic line emission from the neutral (filled triangle) and singly ionized Ar are indicated. The inset shows the SEM image of the Au bowtie structure with a 500 nm scale bar [144].

Various models have been developed to analyze and theoretically explain the HHG process in SP fields [137], [138], [139], [140], [141], [142]. Ciappina et al. presented a quantum mechanical model to explain the increase in the bowtie-based HHG cut-off [143]. The laser electric field results spatially inhomogeneous in the bowtie structure gap, which modifies the electron trajectories and increases the HHG cut-off. The inhomogeneous field distribution in the gap of the bowtie structure is calculated using a 3D finite-element method. The electron motion was calculated using a dimensionally reduced (1D) time-dependent Schrödinger equation (TDSE) based on the calculated field distribution. Figure 9a shows the HHG spectrum from the bowtie nanostructure calculated using the 1D TDSE. Black and red lines indicate the HHG spectra driven by spatially homogeneous and inhomogeneous fields in the bowtie gap. The left inset in Figure 9a shows the calculated electric field *E*(*x*, *t*) as a function of the position when the gap center is at *x* = 0. The solid line represents the calculated data from the finite-element method; the red dashed line represents the nonlinear fitting data. The right inset in Figure 9a shows the intensity enhancement distribution near the bowtie gap. According to the calculated HHG spectrum, the HHG cut-off in the inhomogeneous field is amplified significantly compared to that of the homogenous field. This enhancement is attributed to the electron motion confinement and the modifications in the electron trajectories (increase in the acceleration), due to the parabolic shape of the driven field.

After several plasmonically enhanced gas-phase HHG studies were conducted, a few issues were raised in terms of their reproducibility, efficiency, and ambiguity between atomic line emission and HHG [144–147]. Sivis et al. presented measurements with a very similar setup as to the first SP-enhanced gas-phase HHG experiment employed by Kim et al.; they mentioned that the measured spectrum was an incoherent gas atomic line emission, not coherent HHG.

The experiment was induced by focusing an 8 fs pulse on Ar gas in the presence of a bowtie nanostructure array. Figure 9b shows the measured spectral density. The black line represents the first diffraction order signal, and the red line represents the second diffraction order signal with a higher resolution. Triangles represent the atomic line emissions of Ar gas, and hollow and filled triangles indicate each single ionized and neutral ionized atomic line emissions, respectively. An agreement between the measured harmonic spectral and Ar atomic line emission indicates that the generated XUV spectrum from the bowtie plasmonic structure setup is the incoherent atomic line emission of the Ar gas. Besides, the aforementioned 3D metal waveguide HHG spectra showed the coexistence of coherent high harmonics and atomic line emission. In SP-enhanced HHG, ambiguity, efficiency, and reproducibility issues exist because the nanoscale localized electric field around the plasmonic structure interacts with a small number of sparsely existing gas atoms, which substantially reduce the number of emitters and, consequently, the HHG photon flux.

#### 2.2.3 HHG from solids

Ghimire et al. first demonstrated HHG from bulk crystals (ZnO) using a mid-infrared ultrafast laser [148]. Compared to gas atoms, solids comprise a denser and periodic array of atoms and allow a relatively large number of atoms to participate in the HHG process. Thus, the HHG in solids has a higher conversion efficiency and can be induced with relatively low intensity $\left(1.0\times 10^{12}\;\text{W}/{\text{cm}}^{2}\right)$ laser sources. Solid interatomic bonds form overlapped energy bands of atoms, which creates a unique electron band structure of the solid [133, 149], [150], [151], [152], [153], [154], [155], [156], [157]. The solid HHG process depends on the electron band structure and cannot be well described using the three-step model. Up to now, various models have been proposed to interpret and explain the solid-HHG mechanisms, considering they appear to be more complicated than gas-phase HHG [158], [159], [160], [161], [162].

**Figure 10:**
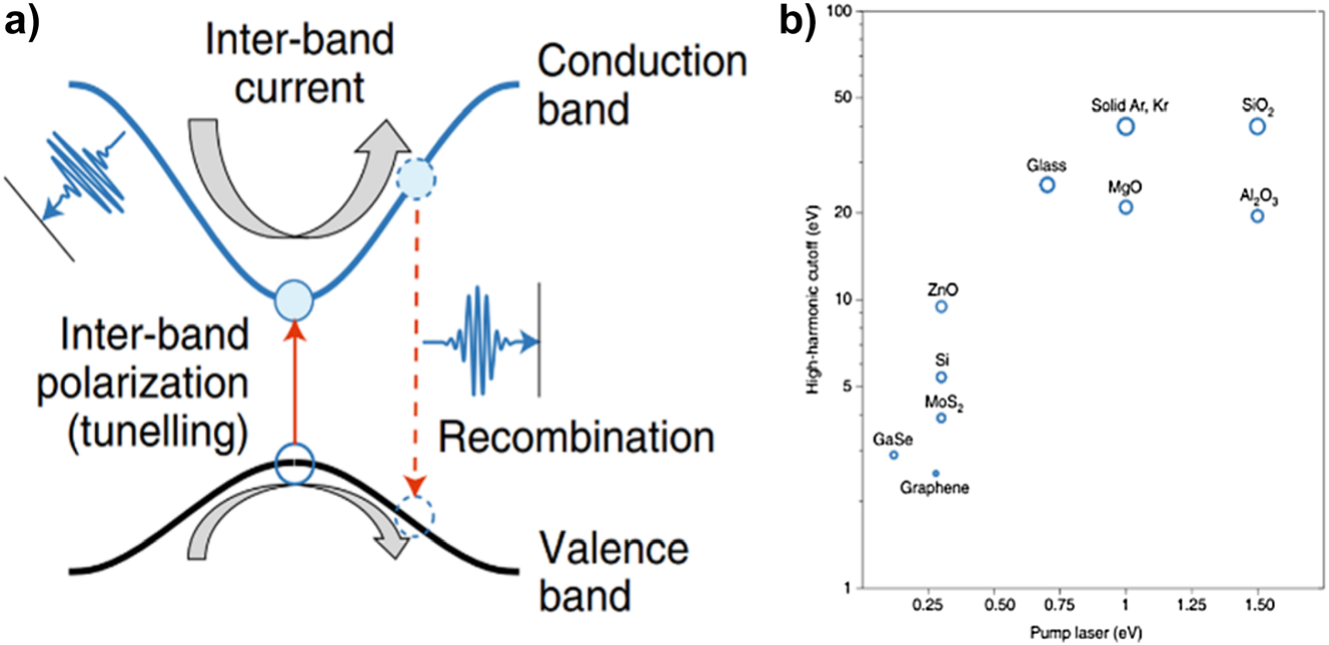
HHG in solids. (a) Mechanism of HHG in solids: momentum-space interpretation. Under strong field conditions, electrons in the solids ascend from the valence band to the conduction band by tunneling effect. Electron–hole pairs are accelerated along with the vector potential, and the intraband HHG is attributed to the interband current in the conduction band, whereas the interband HHG depends on the electron recombining with its left-behind hole (interband recombination). (b) HHG in various solid materials. The high harmonic cut-off depends on the band structure of the solid and the wavelength of the pump laser. The large bandgap dielectric materials enable higher-order harmonics because of the high damage threshold at a short wavelength, whereas the narrow bandgap materials such as semiconductors or 2D materials require a relatively longer wavelength for the HHG to develop [150].

Figure 10a illustrates the momentum-space interpretation of the solid HHG mechanism: electrons in the valence band are excited into the conduction band when a strong field irradiates the solid. Excited electrons are accelerated along with the conduction band by the laser field (intraband transition), and they recombine with the left-behind holes in the valence band (interband transition). Figure 10b shows how the HHG cut-off energy scales with the pump laser photon energy (eV) from various solid materials. Because the HHG process in solids is influenced by the intrinsic electron band structure, an appropriate pump laser has been used for each solid material. For example, relatively long wavelengths are required for semiconductors or 2D materials with a small bandgap, whereas short wavelengths are required for dielectrics with a large bandgap [150, 163–165].

#### 2.2.4 SP-enhanced HHG from solids

**Figure 11:**
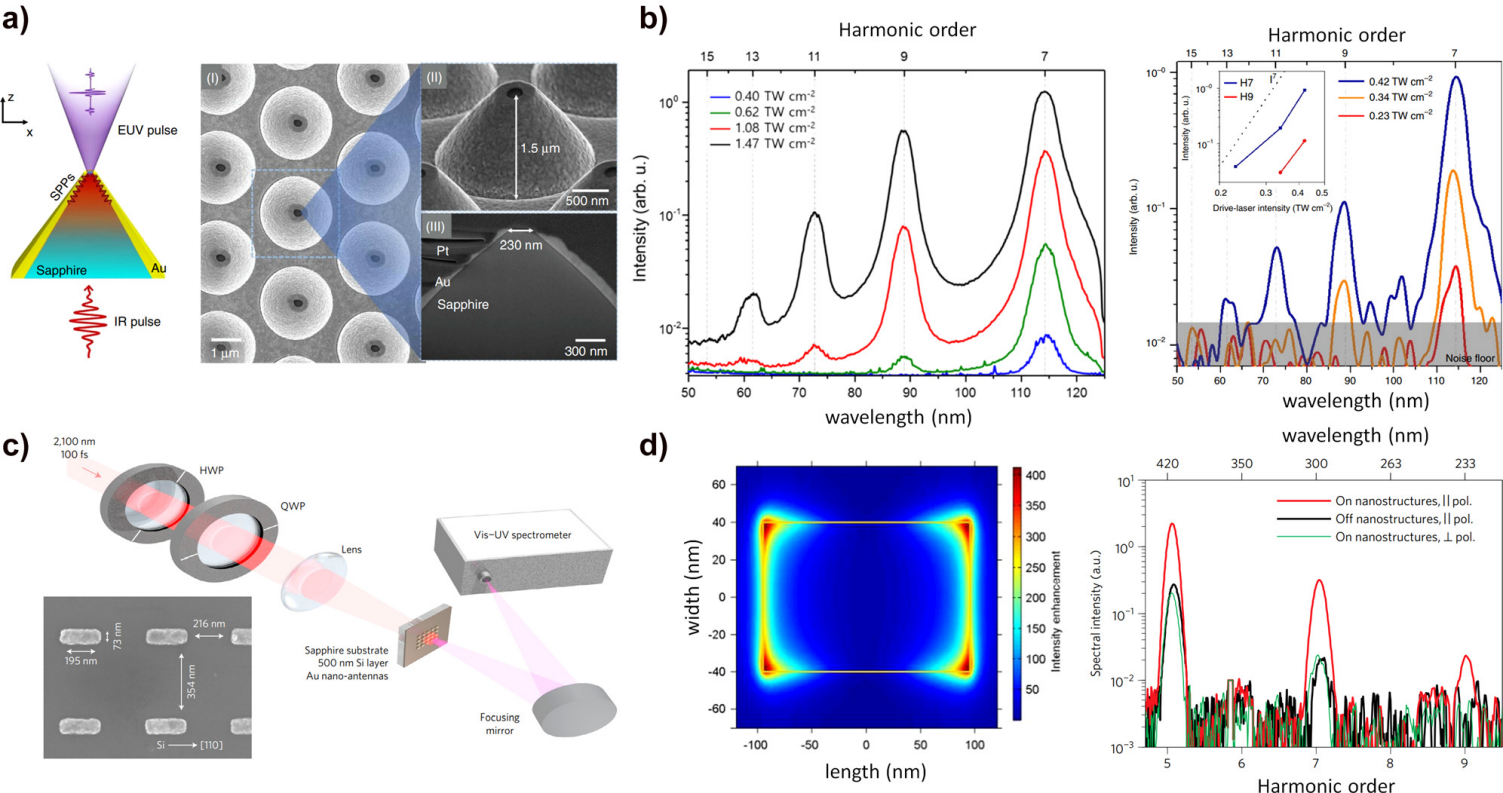
Plasmonically enhanced HHG in solids. (a) Schematic of plasmonically enhanced HHG in an Au-sapphire funnel structure. The SPP propagates along with the Au-sapphire interface and localizes at the tip, which reaches enough intensity for the HHG to happen. The right figure shows the SEM image of the fabricated Au-sapphire funnel structure. (b) HHG spectrum from the bulk c-plane sapphire (left) and plasmonic sapphire funnel structure (right). The plasmonic sapphire funnel structure generates higher-order harmonics (13th order) compared to bulk sapphire (7th order) at a similar input laser intensity ( $∼0.4\times 10^{12}$ W/cm^2^) [165]. (c) Schematic of plasmonically enhanced HHG from silicon using an Au nanoantenna array setup. SEM image of an Au nanoantenna array. (d) Numerically computed intensity distribution around the Au nanoantenna at a polarization parallel to the nanoantenna’s major axis (*x*-direction). The high harmonic spectrum from the Au nanoantenna array with different polarization conditions. The HHG spectrum from the Au nanoantenna array with parallel polarization (red line) and HHG spectrum without the nanoantenna with the same polarization condition (dark blue line). The HHG yields do not increase at the nanoantenna nonresonance condition (green line) [166].

Because SP-enhanced HHG in solids can have more interacting atoms in the local plasmonic field than gas-phase HHG, SP-enhanced HHG in solids using various plasmonic structures has been reported recently. Han et al. demonstrated a SP-enhanced solid-HHG with a cone-shaped Au-sapphire nanostructure [165]. Figure 11a shows a schematic of the SP-enhanced HHG process in the Au-sapphire nanostructure (left) and a scanning electron microscope (SEM) image of the structure (right). When the nanostructure is irradiated by the laser field, the SPP is induced at the Au-sapphire interface and propagates to the tapered tip. It was numerically calculated that approximately a field enhancement of 10^2^ times occurs at the tip by focusing the SPP field through the tapered wall. The HHG was induced by focusing an NIR laser pulse (12 fs, 800 nm, and $0.42\times 10^{12}$ W/cm^2^) through the bottom of the nanostructure.

Figure 11b (left) shows the HHG spectrum at 0.40, 0.62, 1.08, and $1.47\times 10^{12}$ W/cm^2^ peak intensities in the bulk sapphire. Figure 11b (right) shows the HHG spectrum at 0.23, 0.34, and $0.42\times 10^{12}$ W/cm^2^ peak intensities in the Au-sapphire nanostructure; the inset shows the driving laser intensity dependence on the high harmonic yield at the similar light intensity ( $0.40\times 10^{12}$ W/cm^2^ with bulk sapphire, $0.42\times 10^{12}$ W/cm^2^ with plasmonic structure), Au-sapphire nanostructures support a higher cut-off in HHG (13th) than the bulk sapphire substrate (7th) at the same input intensity. This means that the plasmonic structure increases the solid HHG yield and cut-off by the locally enhanced ultrafast EM field.

Vampa et al. demonstrated an SP-enhanced HHG with a plasmonic antenna array [166]. Figure 11c shows a schematic of the experimental setup; the inset shows the SEM image of the plasmonic nanoantenna. The HHG was induced by focusing an NIR laser (100 fs with a center wavelength of 2100 nm, and $0.03\times 10^{12}$ W/cm^2^ peak intensity) to the nanoantenna array. Figure 11d (left) shows the numerically calculated intensity enhancement of the plasmonic nanoantenna. A localized SP is induced when the incidence beam polarization follows the nanoantenna major axis (*x*-axis); a maximum intensity enhancement of 400 was numerically calculated. Figure 11d (right) shows the measured HHG spectrum when the polarization of the incident beam is parallel (red line, resonance) and perpendicular (green line, off-resonance) to the major axis of the antenna. Further, the black line represents the bulk silicon HHG spectrum. At the same laser intensity, HHG spectra are measured up to the 9th order in resonance, and up to the 7th order in off-resonance and bulk silicon. They confine the plasmonically enhanced area to as much as 20×20 nm^2^ near the antenna edge, and at that region, $3\times 10^{3-4}$ times enhanced HHG emission yields are estimated. Thus, the plasmonic nanoantenna on silicon extends the HHG cut-off and improves the HHG yield by 5–10 times, though the nanoantenna occupies only 8% of the sample’s surface area.

**Figure 12:**
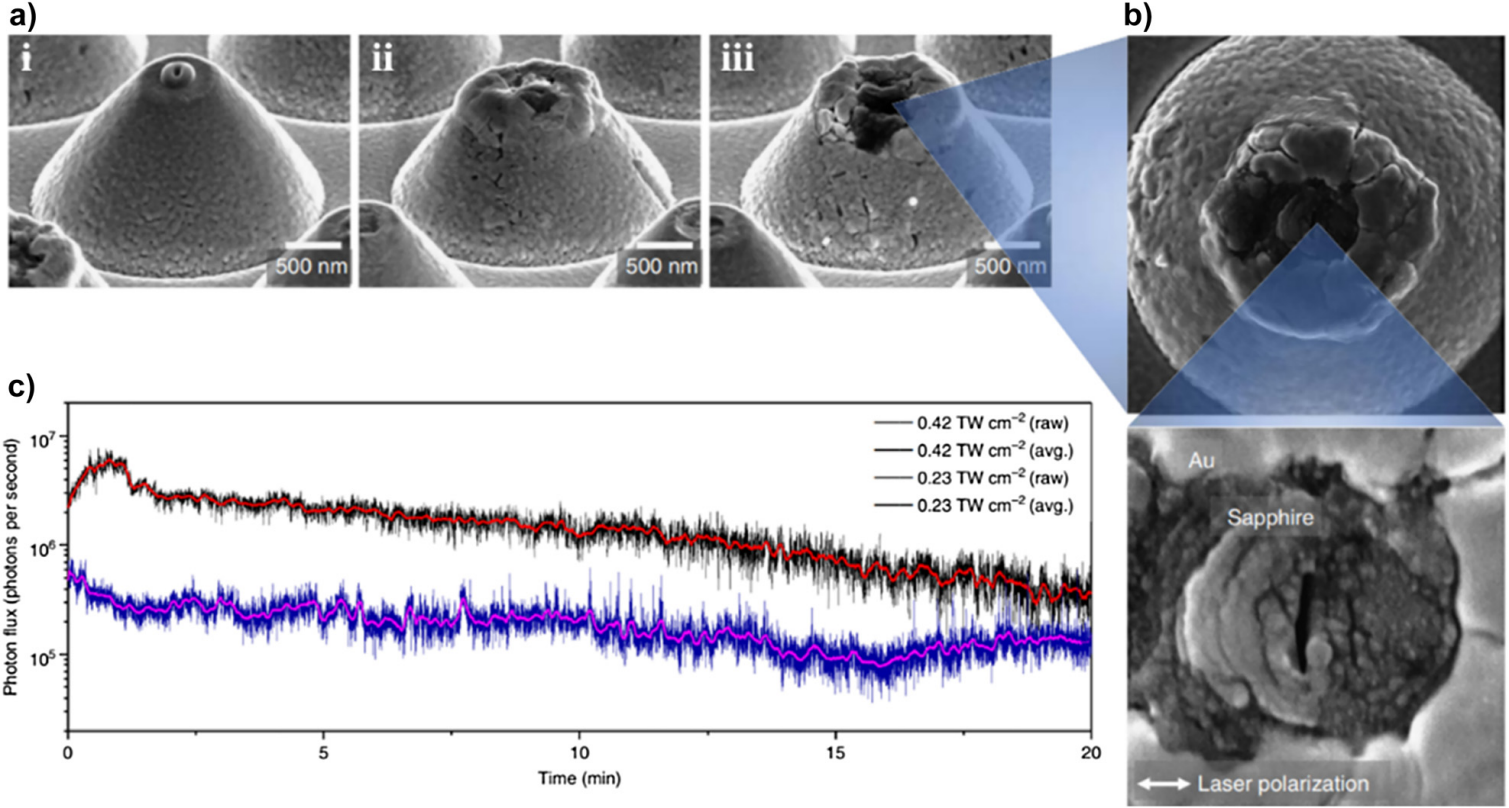
Laser-induced damage in plasmonic nanostructures. (a) SEM image of the laser-induced damage in an Au-Sapphire funnel structure for an incident intensity of (i) 0.23, (ii) 0.42, and (iii) $0.66\times 10^{12}$ W/cm^2^. (b) Magnified top view of a deformed Au-Sapphire funnel structure for $0.66\times 10^{12}$ W/cm^2^. (c) Time-dependent variation of the XUV photon flux (H7, H9, H11, and H13) from an Au-sapphire funnel structure at two different input laser intensities [165].

The generated SP increases the HHG yield and cut-off in solids; however, the accumulated heat in the plasmonic structure can deform its original shape [165], [166], [167]. Figure 12a shows the SEM image of the thermally deformed Au-sapphire nanostructure after being irradiated at various laser intensities (0.23, 0.42, and $0.66\times 10^{12}$ W/cm^2^); the thermal deformation increases with the incident intensity. Figures 12b shows a significant deformation in the Au and sapphire near the tip; these damaged structures cannot effectively focus the SPP. Figure 12c shows the time dependence of the XUV photon flux at 0.23 and $0.42\times 10^{12}$ W/cm^2^ incidence intensities. A steady degradation occurs over time, and a noticeable deterioration is confirmed at a relatively high incidence intensity ( $0.42\times 10^{12}$ W/cm^2^).

### 2.3 Ultrafast photoelectron spectroscopy with plasmonics effect

In 1905, Einstein proposed a theory for the photoelectric effect using a concept first put forward by Max Planck, who suggested that light consists of photons. In classical optical theory, the photoelectric effect is defined as the emission of freed electrons from a material when an illuminating photon has higher energy than the work function of such material and the energy of the emitted electron only increases if the incident light increases. Later, the development of advanced lasers helped realize a nonlinear photoelectron emission process that was previously impossible. In the nonlinear photoelectric effect, the Keldysh parameter is applicable to distinguish between the multiphoton (photon picture) or tunneling (wave picture) regimes. The emitted photoelectron from the target sample possesses information of the material properties and the strength of the local field that accelerates it [168], [169], [170]. For example, it is possible to estimate the field strength or distribution near the surface of the target sample by analyzing the kinetic energy of the emitted photoelectrons. In photoelectron spectroscopy, the spatial distribution of emitted photoelectrons is achieved by exploiting the microchannel plate (MCP) properties, and the energy is measured by time of flight-based methods. However, the spatial resolution of the MCP without a magnetic lens-based imaging system is low and unable to resolve the nanometer scale distribution of the emitted photoelectrons. This issue can be fixed by employing a one-dimensional structure like a sharp tip for the target sample. Some researchers demonstrated strong field effects by using a tungsten sharp tip without pulse amplification by taking advantage of the lightning rod effect [171, 172]. Later, thanks to advanced fabrication and chemical synthesis techniques, various plasmonic structures were applied to photoelectron emission experiments. Here, it would be possible to exploit the plasmonic characteristics to design sensors or well-tailored electron sources, such as laser-triggered sources of ultrashort electron pulses, which are used in electron interferometry and carrier-envelope phase detection [16, 173]. The plasmonic effect further localizes the electric field and this allows a better spatial resolution in photoelectron spectroscopy. Additionally, this localization increases the field intensity in such a way that it is now possible to distinguish the plasmonic-field driven photoelectrons by analyzing their kinetic energy. In this section, an SP-based photoelectron emission study [174] and a carrier-envelope phase (CEP) detector [175], [176], [177], [178], [179] are introduced.

#### 2.3.1 SP-enhanced photoemission

**Figure 13:**
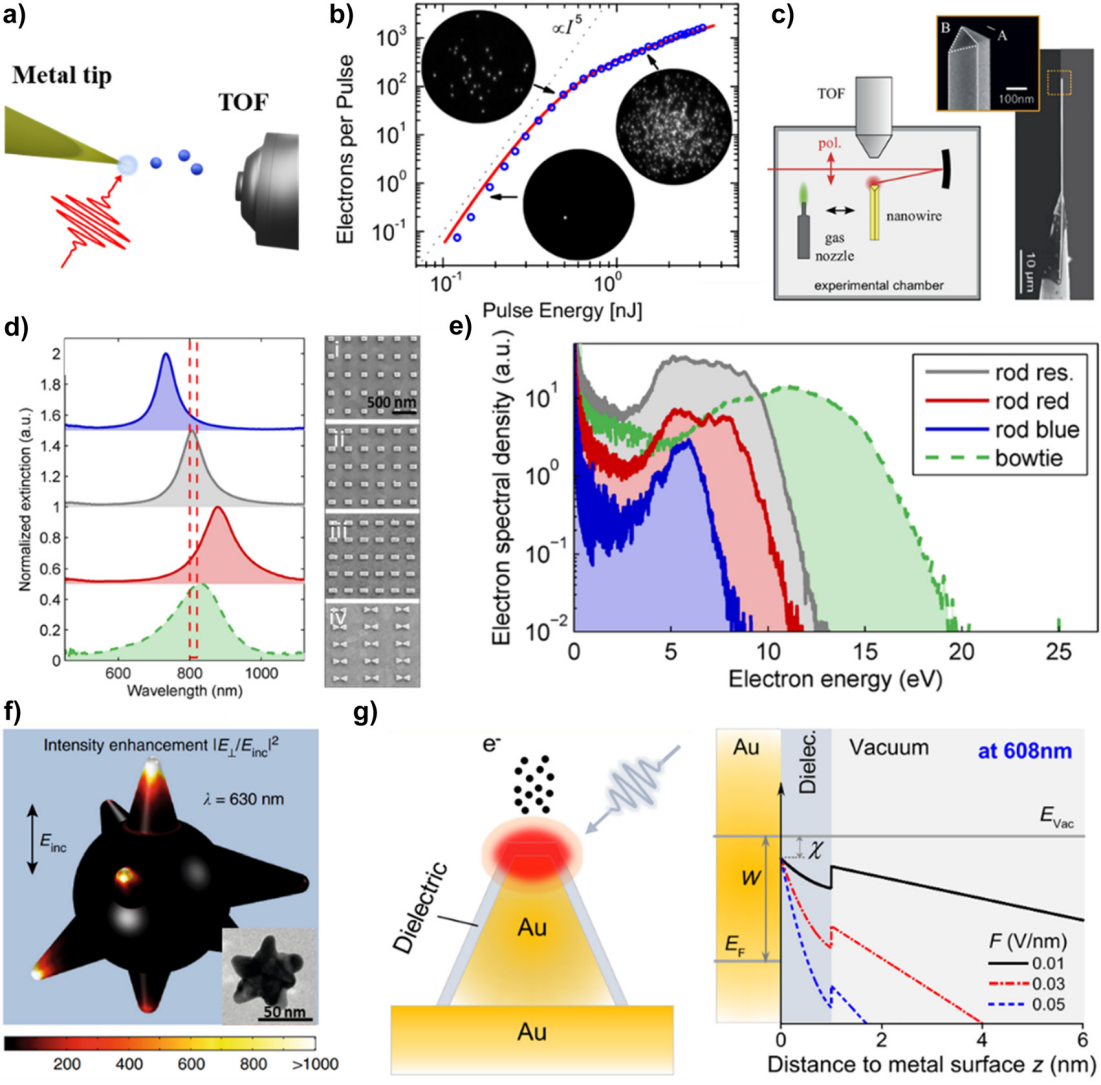
Plasmonically enhanced photoelectron emission. (a) Schematic of photoelectrons emitted from a sharp metallic tip by taking advantage of the plasmonic resonance, analyzed by a time-of-flight electron (TOF) spectrometer. The strong plasmonic field accelerates the emitted photoelectrons and tailors their dynamics [180]. (b) Pulse energy dependence tip-enhanced photoelectron yields per pulse as a function of the input pulse laser energy (blue circle). The strong field effect induces an abrupt transition with the 5th power-law line (black dashed line). Adapted with permission from ref. [181]. Copyright 2010, APS (c) Schematic of photoelectron emission from single-crystalline Au nanowires, for the electrons with higher kinetic energy. SEM image of single crystalline Au nanowires. Adapted with permission from ref. [182]. Copyright 2017, AIP. (d) Measured extinction spectra and SEM images for nanorods with dimensions of (i) 120 × 87 × 40 ${\text{nm}}^{3}$ , (ii) 152 × 87 × 40 ${\text{nm}}^{3}$ , (iii) 183 × 87 × 40 ${\text{nm}}^{3}$ , and for a bowtie structure with dimensions of (iv) length of 260 nm, a width of 90 nm, and height of 40 nm [183]. (e) Electron spectra for different nanostructure geometries and for a laser peak intensity of $25.1\times 10^{9}$ W/cm^2^. Adapted with permission from ref. [183]. Copyright 2013, ACS. (f) numerical simulation of the localized near-field distribution of a single nanostar upon vertical polarization. TEM image of a single nanostar with a 50 nm scale bar [184]. (g) Schematic of enhanced photoemission from an ultrathin dielectric coated Au pyramid structure. Potential barrier profile of a dielectric coated Au pyramid structure with different external fields (F). Adapted with permission from ref. [185]. Copyright 2020, ACS.

An increase in the photoelectron emission efficiency and spatial control was demonstrated by generating LSPR and SPP in various plasmonic structures. Figure 13a shows a schematic of the photoelectron emission from a plasmonic Au nanotip [180]. When an ultrafast laser is focused on an Au sharp tip, the electric field is highly confined to the tip by the plasmonic resonance. The localized electric field not only produces nonlinear photoelectron emission at relatively low input intensities but also has a high spatial resolution like a point source, which enables a variety of analyses and applications using photoelectrons. Figure 13b shows the SP-enhanced photoelectron yield from the Au nanotip with increasing laser energy [133]. The work function (∼5 eV) of the sharp Au tip requires a 4-photon process for an NIR pulse (∼1.5 eV); however, the electron yield follows a 5-photon process with an increase in the pulse energy. This result indicates that the plasmonic effect of the Au tip affected the nonlinearity of the photoelectron emission. Further, an abrupt transition is observed around 0.6 nJ, in that the photoelectron yields follow the strong-field approximation. The strong plasmonic effect enables the generation of high-energy photoelectrons up to 160 eV from sharp Au single-crystalline nanowires [181]. Figure 13c shows the photoelectron emission experimental setup using a single-crystalline Au nanowire joint with their SEM images [182]. Thanks to the chemically synthesized sharp edges on the nanowire, high-energy electrons originate from the field-induced rescattering in the enhanced nanolocalized fields at the apex of the nanowire.

Conceptually, photoelectron emission from plasmonic structures has freedom in the design geometry, unlike tungsten nanotip-based photoemission spectroscopy. Advanced fabrication technology enables the control of plasmonic properties by tailoring the nanostructure geometry. Dombi et al. demonstrated the manipulation of photoelectrons by designing nanorods with different plasmonic resonance conditions. The evanescent field of the SP around the plasmonic nanostructure affects the acceleration of the emitted photoelectrons, which results in different electron kinetic energy distributions, as shown in Figure 13d and e [183]. Figure 13d (right) shows SEM images of different nanostructures; each resonance wavelength of the nanostructure is confirmed by measuring the optical extinction spectra (left). A relatively high electron kinetic energy cut-off is measured at the structure that matches the resonance wavelength with the incident femtosecond pulse wavelength as shown in Figure 13e. Further, the bow-tie structure enhances even further the field at the gap than single nanorods, letting higher energetic photoelectrons to appear. This result indicates that the strong SP generated in the resonance condition modifies the dynamics of emitted electrons.

The spatial localization and efficiency of SP are significantly affected by the geometry and sharpness of the nanostructure. Despite employing advanced fabrication techniques, the manufacturing of nanoscale structures continues to remain a great challenge. To cope with that, enhanced photoelectron emission from chemically synthesized Au nanostars, with 5 nm-scale tips has been reported [184]. Figure 13f and the inset show the simulated localized enhancement distribution and transmission electron microscope (TEM) images of Au nanostars. The simulation results indicate an intensity enhancement factor exceeding 1000 at the nanostar tip. The photoelectron emission was measured by focusing a CW laser with low intensity, $1.0\times 10^{6}$ W/cm^2^, on the Au nanostars. This result implies that an over 1000-fold intensity enhancement occurred at the 5 nm-scale nanostar tips. In addition, a 3rd order multiphoton process was measured in the Au nanostars. These findings indicate that photoelectron emission can be obtained from low-intensity CW laser-induced strong plasmonic near-field enhancement conditions, when not ultrafast pulse lasers with a strong peak intensity are available.

It was shown that higher energetic photoelectrons can be generated using plasmonic photoelectron emission. There are, however, some limitations, such as their low quantum efficiency. Recently, a theoretical work about enhanced photoelectron emission yield was reported using plasmonic confinement and double tunneling barrier effects in dielectric-coated Au pyramid structures [185]. Figure 13g (left panel) illustrates the enhanced photoelectron emission from a dielectric-coated Au pyramid structure. The generated plasmonic field at the Au pyramidal structure-dielectric interface, localized in the dielectric film; induces a confinement effect. Figure 13g (right panel) shows that the Au-dielectric–vacuum potential barrier dramatically decreases as increasing the incident field strength. Therefore, the dielectric-coated Au pyramid enables studies at a transition regime, i.e. in the region between multiphoton to tunneling, with 10 times lower incident intensity than that without coating.

**Figure 14:**
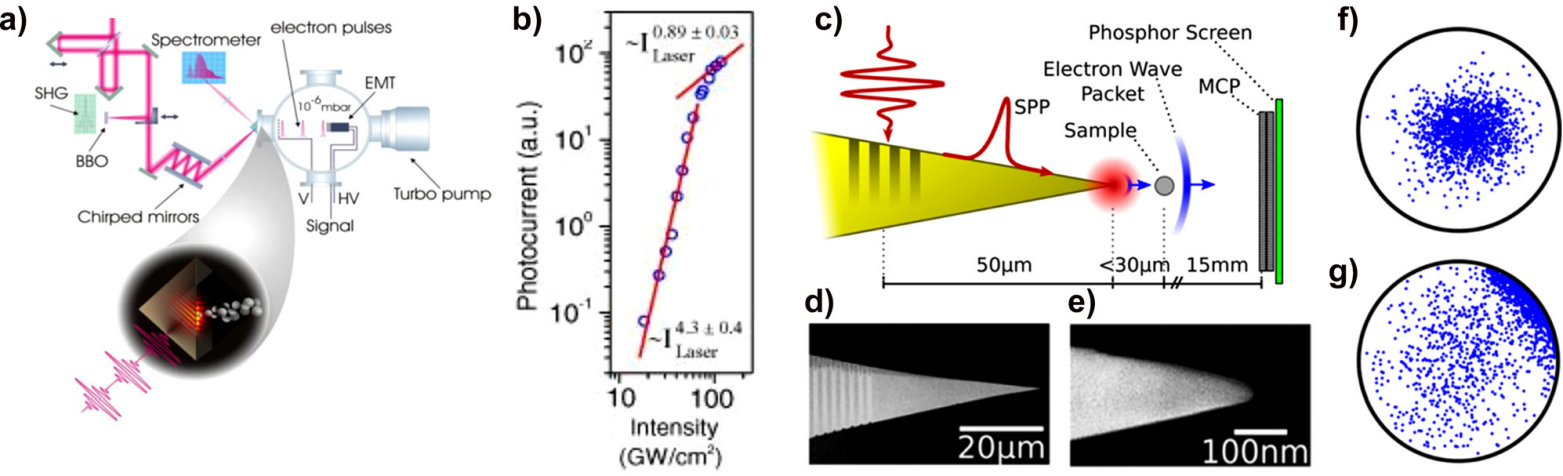
SPP-based photoelectron emission. (a) Schematic of a photoelectron emission experiment setup using the Kretschmann configuration. (b) Intensity dependence of the SPP enhanced photocurrent. Adapted with permission from ref. [189]. Copyright 2010, OSA.(c) Schematic of an electron emitted from a grating sharp nanotip. The illuminated grating generates an SPP, which propagates through the sharp nano taper apex and plasmonically enhances the photoelectron emission. The SEM image of (d) a grating sharp nano taper, (e) a magnified image of a grating sharp nano taper. Spatial distribution of emitted photoelectron emission from (f) a grating sharp nano taper (radius 12 nm), (g) a blunt nano taper (radius 50 nm). Adapted with permission from ref. [190]. Copyright 2015, ACS.

For separating the local plasmonic field from the pump laser field in photoelectron emission, SPP-based photoelectrons could be a better choice than LSPR-based experiments because SPP propagates along with the interface and focus on the tapered geometries [186], [187], [188]. Figure 14a shows the experimental setup of SPP-enhanced photoelectron emission, which SPP generated at a silver/vacuum interface with the Kretschmann configuration [189]. The generation of SPP was confirmed through changes in the reflectivity of the incident femtosecond pulse at the prism and the photoelectron emission yield. Figure 14b shows a focused intensity-dependent measured photocurrent emitted from the silver surface. The photoelectron yields follow a fourth-order power-law at low intensities; this means that a perturbative process occurs below certain intensity and the photocurrent then follows a 0.89 order of power-law, which means the nonperturbative process dominates. The transition of the perturbative to the nonperturbative regime occurs at a considerably lower intensity (<10^11^ W/cm^2^) at the bulk metal film; this implies that SPP significantly enhances the local field, and the photoelectron emission takes place then at the tunneling regime.

It is possible to spatially manipulate SPP by using a grating on a sharp nanotaper structure. Figure 14c illustrates a propagating SPP gathered at the apex of a grating sharp Au nanotaper [190]. The generated SPP at the grating region propagate at a distance of 50 μm and concentrate in the apex, which then shows a photoelectron emission yield 50 times larger than the direct apex focusing photoelectron emission. The sharp Au nanotaper was produced using a chemical etching method, and the grating coupler was fabricated with a focused ion beam method. Figure 14d and e respectively shows the prepared grating sharp Au nanotaper SEM image and an enlarged sharp taper SEM image. The grating sharp nanotaper has an apex radius of curvature of 12 nm. The spatial distribution of the photoelectron emission from the sharp taper was characterized with a microchannel plate detector. Figure 14f and g respectively, shows the spatial distribution of the photoelectron emission from a sharp nanotaper (radius 12 nm) and a blunt taper (radius 50 nm). The photoelectron emission spatial distribution from the sharp nanotip has a fine circularly symmetric pattern, in contrast to the blunt taper, where a partial cone shape is observed. This result means that efficient nanofocusing was demonstrated at the apex of the grating sharp nanotaper. Besides the above-mentioned research, various SPP-based photoelectron emission investigations have been conducted, especially once the SP-enhanced photoelectron emission proved to be a useful tool to directly measure the plasmonic field enhancement [191, 192].

#### 2.3.2 Carrier-envelope phase detector with photoelectron emission

**Figure 15:**
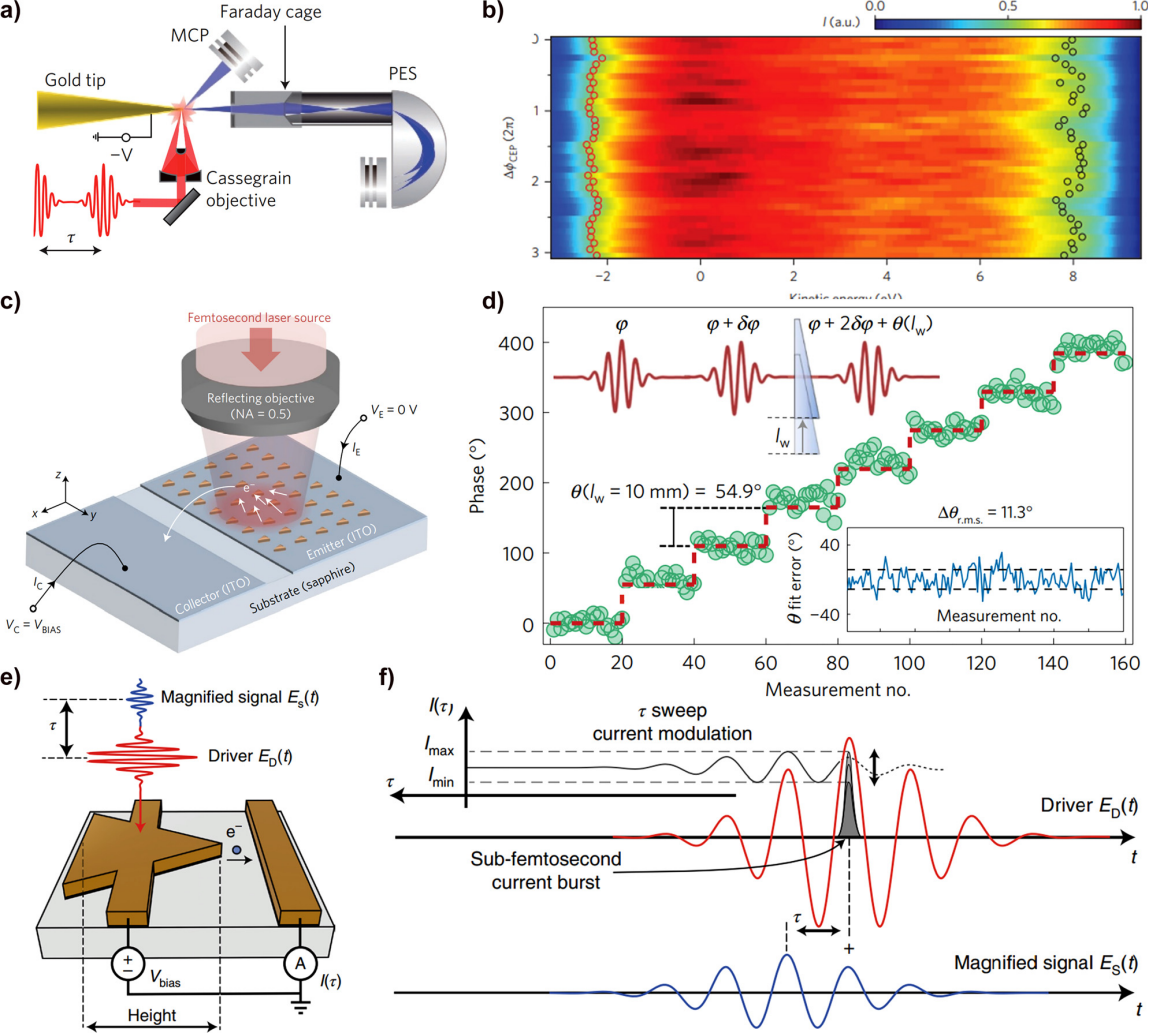
CEP and light-wave dependent photoelectron emission in a plasmonic nanotip under the strong-field condition. (a) Photoelectron emission measurement with a CEP-stabilized few-cycle pulse. The Au tip is electrically biased to modify its potential barrier. Photoelectron yields and kinetic energy are measured with a microchannel plate (MCP) and photoelectron spectrometer (PES), respectively. (b) The CEP-dependent electron kinetic energy spectra under strong field conditions. Red and black circles indicate the low and high energy cut-off, respectively [195]. (c) Schematic of a nanostructure-based optoelectronic device for phase-sensitive photoelectron emission measurement. The device comprises a collector and emitter region with a 5 μm gap. The Au nanostructure array is fabricated on the indium-tin-oxide (ITO) emitter region. The SP effect and external bias allow the device to measure the emitted photoelectron from the nanostructures in ambient conditions. (d) CEP-dependent phase measurement from the device under strong field conditions. The CEP changes by inserting an optical wedge and are measured using the lock-in current detection method. The right inset shows the stability of the measured signal [196]. (e) Schematic of the subcycle field sampling device. The device comprises an Au antenna and nanowire with a 50 nm gap. The emitted photoelectrons in the Au antenna are collected by the Au nanowire, which results in the photocurrent with a lock-in detection method. (f) The schematic of the optical field sampling process. A strong driving field generates a photoelectron with a sub-femtosecond current burst (gray pulse); the photoelectron yields are modulated by the weak field (blue line), which allows the waveform reconstruction [197].

The CEP control of few-cycle pulses affects the photoelectron kinetic energy spectra emitted from metallic nanotips [193]. The emitted electrons are directionally accelerated when the decay length $\left(l_{\text{F}}\right)$ of the optical near-field at the tip is smaller than the quiver amplitude $\left(l_{\text{q}}\right)$ of the electron $\left(\delta =l_{\text{F}}/l_{\text{q}}<1\right)$ , in the strong-field regime where the above-threshold ionization (ATI) and above-threshold photoemission (ATP) processes occur [192, 194]. This implies that CEP effects can be observed at the Au tip, where large field enhancements are achieved [193]. Figure 15a shows a photoelectron emission experiment setup with a CEP-stabilized femtosecond pulse and an Au tip. Figure 15b shows the change in the photoelectron’s kinetic energy spectra with the CEP-variation at the strong-field regime. Here, red circles indicate a low cut-off and the black circle indicates a high cut-off. There is a noticeable change in the kinetic spectral energy cut-off as a function of the CEP. This implies that a change in the waveform, caused by the CEP, affects the near-field amplitude at the Au tip; this, in turn, modifies the electron acceleration within the localized-field gradient.

A chip-scale optoelectronic CEP detector, using the waveform sensitivity of the photoelectron emission from a plasmonic metal structure in an ambient environment, was reported recently [196]. Figure 15c shows the layout of the device used in this experiment. There is a 5 μm gap between the emitter and collector regions, and the emitted electrons are carried away by the bias voltage when the fs laser is focused on the nanostructure. The generated electrons have a mean free path of several hundred nanometers under nonvacuum conditions; however, LSPR and the additional dc bias allow them to transit over the 5 μm gap. Figure 15d shows the CEP-dependent phase measurement results retrieved from the photoelectron detection. Through a lock-in current measurement, it is confirmed that the phase of $f_{\text{ceo}}$ responds sensitively to changes in the CEP.

Recently, a chip-scale optoelectronic device for low-energy NIR femtosecond pulse measurements was reported [197]. This device uses a sub-optical-cycle sampling technique with a plasmonic structure, and it can be employed under ambient conditions. Figure 15e shows a schematic of the plasmonic photoelectron emission device. The induced localized SPP enables electron emission from the tip and the emitted electrons are collected on the opposite Au wire when the driver field (red) is focused on the Au triangle [198]. Figure 15f shows that photoelectrons are emitted in sub-femtosecond current bursts (red) and the photocurrent modulation is achieved by scanning the time delay with a weak field (blue). The current change is measured while controlling the scanning timing of the driver and weak fields; the converted waveform is then tracked. The on-chip petahertz field-sampling devices are applicable for the time-domain characterization of attosecond electron dynamics and optical-field-driven light–matter interactions.

## 3 Ultrafast plasmonics and ultraprecision spectroscopy

### 3.1 Plasmonic spectroscopy

Plasmonic evanescent fields are strongly confined at a surface, as SPPs, or at a local area, as the LSPR, of plasmonic nanostructures; these fields can be substantially modified by changes in the structural shape and local refractive index induced by the target materials bound at the surface [9]. This high sensitivity provided by the plasmonic effect gave rise to plasmonic spectroscopy, which has been widely used to track atomic/molecular fingerprints at the nanoscale. Therefore, this plasmonic spectroscopy has been widely applied in chemical [199], biological [200], and environmental [201] research fields having enabled high sensitivity, label-free, and real-time measurements [202].

**Figure 16:**
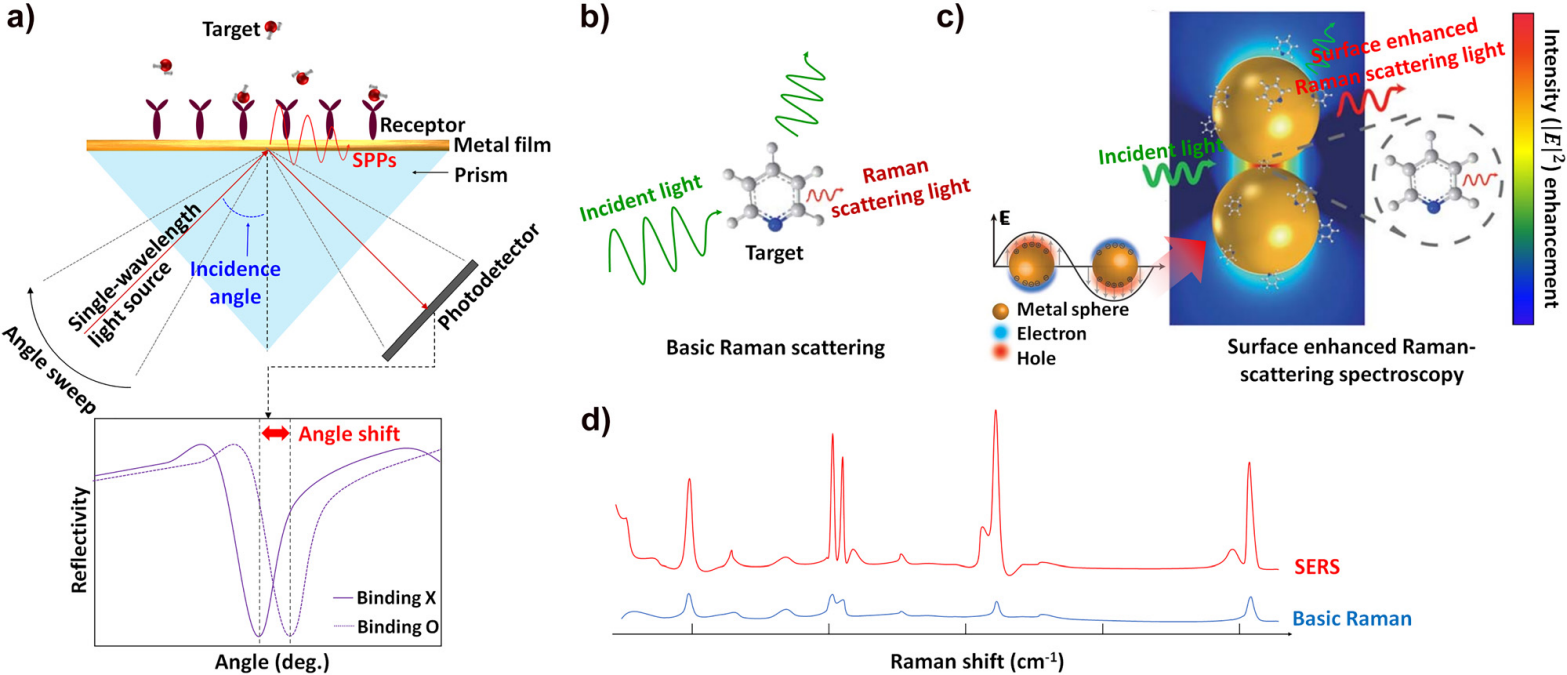
Two representative types of plasmonic spectroscopy. (a) General setup of SPR spectroscopy. The SPR spectroscopy measures the reflected light intensity depending on the angles affected by the refractive index changes on a plasmonic metal surface. The SPP angle can be changed by the biochemical reaction between the target and receptor. (b) Basic mechanism of Raman scattering. The Raman spectroscopy measures the specific scattering light with different wavelengths upon the target molecules. (c) The schematic sketch of SERS. The light is strongly confined through the LSPR of the subwavelength metallic nanoparticles, which enhances the scattered light intensity [212]. (d) The comparison of photon yields for typical Raman spectroscopy and SERS. The effective peak intensity of SERS is much higher than that of the typical Raman spectroscopy.

One representative plasmonic spectroscopy technique is the SPR spectroscopy, which is performed using an experimental apparatus based on the attenuated total reflection (ATR) method, as shown in Figure 16a [9, 203]; it shows the Kretschmann configuration with a prism to induce the SPPs, propagating along with the metal surface (usually Au), which are also used for strong-field photoelectron emission. When the single-wavelength light is directed through a high refractive index prism, the light ray is generally totally reflected at the interface between the metal film and the prism. However, when the momentum of the incoming light is equal to the momentum of the SPPs, by changing the incidence angle, the photons are absorbed by the metal layer, resulting in a reduction of the light intensity in the reflected light. This distinct feature in the reflection at the specific incidence angle implies the SPPs generation. Then, this shadow in the reflected light profile is sensitive to changes in the refractive index occurring on the metal film where the SPP evanescent field exists. This enables molecular sensing such as polymers, DNA, or proteins [9] by monitoring the reflected light intensity, which is induced by binding the target molecules to the receptors on the metal film.

Further, SPR spectroscopy can be conducted through not only metallic flat films but also nanoparticles; the method using nanoparticles is used for monitoring the nanoparticle solution bright color emitted by LSPR. Unlike the ATR method, SPR spectroscopy using plasmonic nanoparticles is very suitable for SPR fluorescence spectroscopy through color sensing, since they have the advantage of obtaining a strong light absorption band, from the ultraviolet to the visible region [204].

The other representative plasmonic spectroscopy is the surface-enhanced Raman spectroscopy (SERS) technique, a well-known plasmonics application. SERS is based on basic Raman scattering spectroscopy technique, as shown in Figure 16b; however, it is a weak nonlinear process, requiring high SNR spectrometers or strong field amplification methods such as LSPR. The SERS utilizes a specific amplification mechanism to monitor the Raman scattering from the molecular vibrations, amplified by a specific process [205]. Before introducing SERS, we need to mention some details about the two known SERS mechanisms. In fact, it is true that SERS was performed by a specific mechanism that can enhance the Raman scattering light [206]; however, the exact mechanism was not identified for a long time. The two most supported mechanisms for SERS are the chemical mechanism [207, 208] and the EM mechanism [209], [210], [211]. Formally, the Raman scattering intensity is proportional to the square of the dipole moment, and it greatly depends on both the molecular polarizability and the EM field. The chemical mechanism is based on the fact that the molecular polarizability is amplified by the interaction between the metal surface and the attached molecules on the surface; therefore, the Raman scattering intensity is higher than that without the metallic materials. In the EM mechanism, the intensity of optical fields is considerably enhanced by LSPR in metallic sub-wavelength structures such as rough surface films and nanoparticles, as shown in Figure 16c and d; the high enhanced-field of the incident light causes Raman scattering to be amplified because the vibrating molecules absorbed on the plasmonic sample obtain sufficient energy. We explain SERS using LSPR in plasmonic samples by following the EM mechanism [209]. Basic Raman scattering spectroscopy has difficulties in accessing single-molecular-scale measurements caused by the effective scattering of the cross-sectional area, which is considerably smaller than the fluorescence region generated by the incident light. In contrast, the Raman scattering intensity in the SERS mechanism is significantly enhanced at the gap of plasmonic nanoparticles by the local EM field amplification induced by LSPR, as depicted in Figure 16c; it provides a sufficient scattering intensity of the signal for single-molecular-scale sensing [213, 214]. Thus, SERS allows a new approach for single-molecular-scale Raman scattering spectroscopy. SERS has made significant contributions to the technical development of surface absorption molecular spectroscopy by enabling high-resolution analysis at a single-molecule level and the real-time monitoring of molecular reactions.

It is possible to overcome the limitations in terms of spectral resolution and precision by applying an optical frequency comb to plasmonic spectroscopy. Plasmonic samples can have various structural shapes such as metallic nanoparticles, nanostructure arrays, and flat surface films, and they can produce plasmonic effects such that an optimized spectroscopy approach can be implemented by selecting an experimentally tailored structural shape. Plasmonically enhanced spectroscopy has accelerated applications in fields such as physics, chemistry, and biology. However, most SPR spectroscopy is based on colorimetric analysis and depends on the measurement of the spectral peak shifts, the intensity variations at the single wavelength of interest, or plasmonically enhanced nonlinear/fluorescence emissions, all of which are based on light intensity measurements. There is a fundamental limitation to spectral intensity measurement; this method is considerably influenced by low-frequency noise, which hinders precision. Therefore, it is necessary to devise new spectroscopic techniques, able to improve the poor performance of colorimetric methods.

### 3.2 Frequency comb spectroscopy

**Figure 17:**
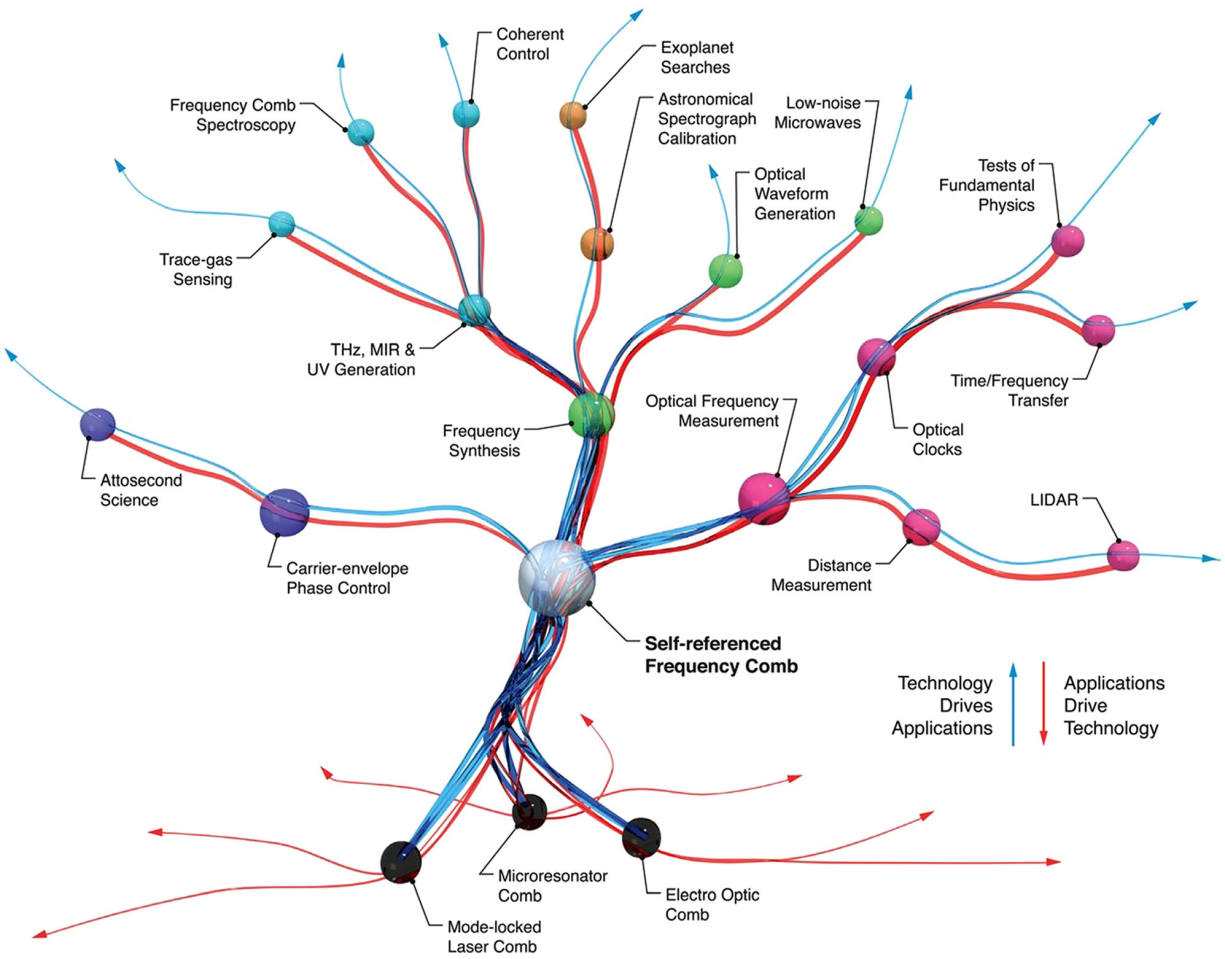
Frequency comb evolutionary tree. The diagram represents an overview of various optical frequency comb applications [215].

The optical frequency comb referenced to an atomic clock is an ultraprecision tool, widely used in diverse fields of length, time, and frequency standard experiments, because of its ability to measure both frequencies and phases very precisely. The development of optical frequency combs rapidly progressed in the past few decades and has spread to broader fields as indicated in the tree diagram shown in Figure 17 [215].

#### 3.2.1 Optical frequency comb generation

**Figure 18:**
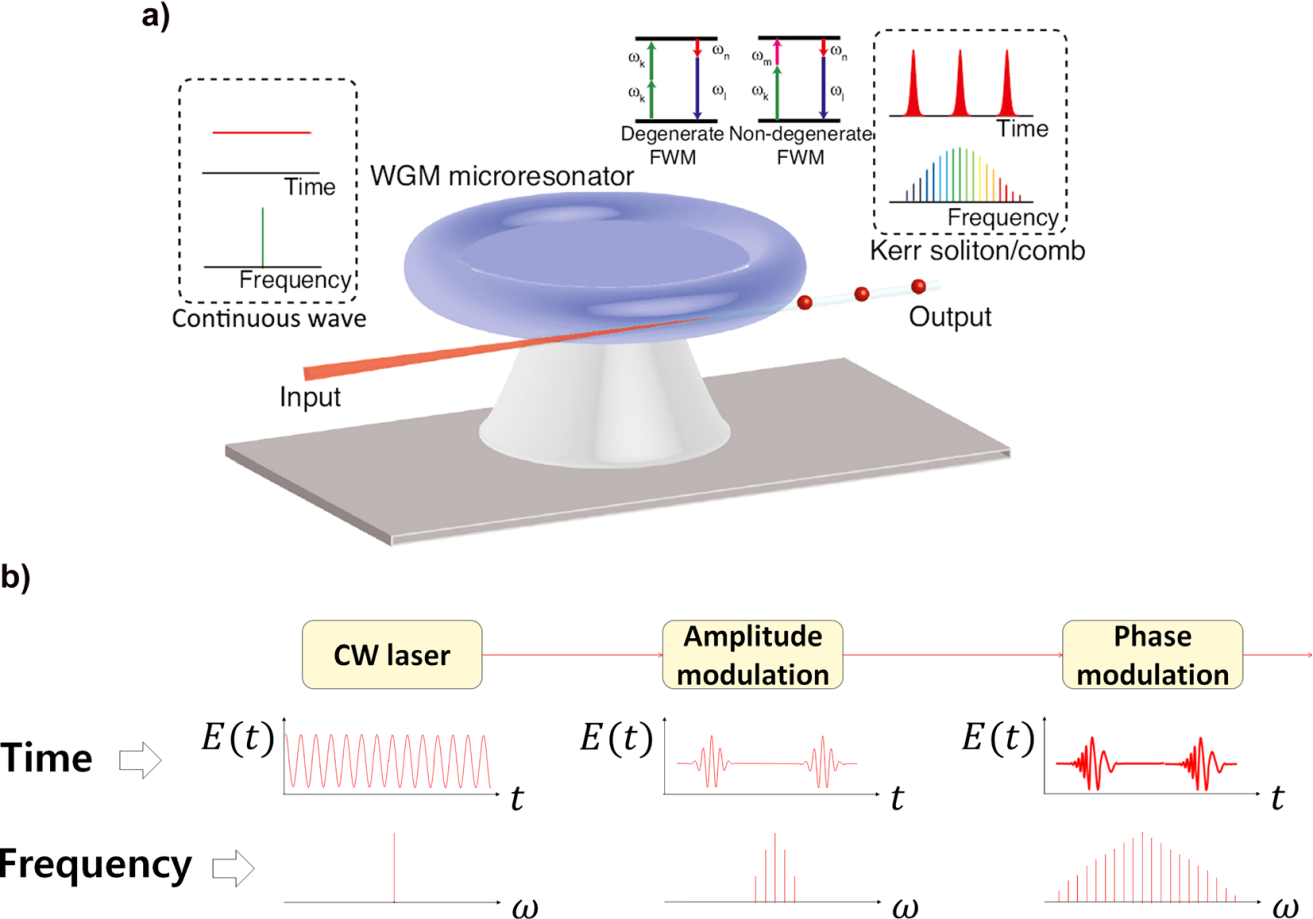
Optical frequency comb generation. (a) A typical 3D sketch of a toroidal cavity, one of the WGM microresonators, and the optical frequency comb generator. The input CW laser is incident on the WGM microresonator and experiences the FWM through the Kerr effect induced by an ultrahigh q-factor of the WGM microresonator and sufficient power of the incident laser [218]. (b) Normal mechanism of the optical frequency comb generation based on optical modulation. The CW laser is modulated by the various types of modulators increasing the optical modes in the frequency domain. This optical frequency comb can be controlled to make a uniform intensity by tuning each optical modulator easily.

An optical frequency comb can be generated by not only the mode-locking technique but also by using various optical apparatuses such as the WGM microresonator [216], [217], [218], [219] and optical modulator [220], [221], [222], as shown in Figure 18a. WGM microresonators have a variety of structural shapes [218] such as microdisks, microspheres, microtoroids, and microrings. All of them can confine the light strongly by internal total reflection at the interface between the medium and air. For optical frequency comb generation via microresonators, a parametric frequency conversion process is performed because of the Kerr nonlinearity [223, 224] generated when a CW laser with sufficient intensity hits on a microresonator with an ultrahigh q-factor; the nonlinear process takes several sidelines positioned uniformly with a specific spacing referenced by the optical frequency of the pump CW laser [216]. Further, the ultrahigh q-factor of the medium leads to an ultralow-threshold, which is the reciprocal of the square of the q-factor at which the nonlinear optical phenomena can occur easily. This results in a nonlinear frequency conversion such as four-wave mixing (FWM) based on third-order nonlinear effects. The frequency conversion is generated continuously according to not only the pump frequency but also the follow-up frequency generated through the conversion, while repeating both degenerate and non-degenerate FWM processes as shown in Figure 18a; the generated optical frequency modes, which become the optical frequency comb, can be produced by following all these processes.

Another alternative method for comb generation is to use an optical modulator, normally EOM, which provides amplitude and phase modulations as shown in Figure 18b. This approach is performed by applying the amplitude and phase modulation sequentially, and the full process is as follows: a CW laser is amplitude-modulated in the form of a pulse envelope, which generates a few sidelines referenced on the CW central frequency. The amplitude-modulated light is then phase-modulated step-by-step; the sidelines with uniform spacing, corresponding to the modulation frequency, can be formed from several prior sidelines generated by the amplitude modulation. It is possible to create numerous well-defined lines, i.e. an optical frequency comb, using this sequential optical modulation by several optical modulators. Recently, chip-scaled optical modulators using sophisticated fabrication techniques [225] and graphene [226, 227] have been actively studied, and this has boosted the development of chip-scaled optical frequency comb devices.

#### 3.2.2 Optical frequency comb spectroscopy

For spectroscopy, the optical frequency comb provides many advantages as a light source having unique optical characteristics such as (1) broad spectral bandwidth, which is good for detecting a variety of molecular species; (2) high spatial coherence, which provides the possibility of obtaining better sensitivity when complex optical systems are used; and (3) narrow linewidth and precision, which are essential for every spectroscopic technique. For atomic or molecular spectroscopy, the target spectral lines are all widely different depending on their types, and therefore, the optical frequency comb needs to be developed over considerably long broadband wavelengths. Therefore, the optical frequency comb generation has been widely explored in broad spectral regions ranging from the terahertz (1 THz = 10^12^ Hz) [228, 229] to the XUV [96, 230, 231]. Finally, it is possible to realize more stable and precise measurements than before by emitting an optical frequency comb in various spectral bands because it can be applied to various targets. Thus, the optical frequency comb spectroscopy can be employed in ultracold molecules [232], human exhaled breath analysis [233], nonlinear coherent spectroscopy [234, 235], and high precision molecular spectroscopy [236, 237].

**Figure 19:**
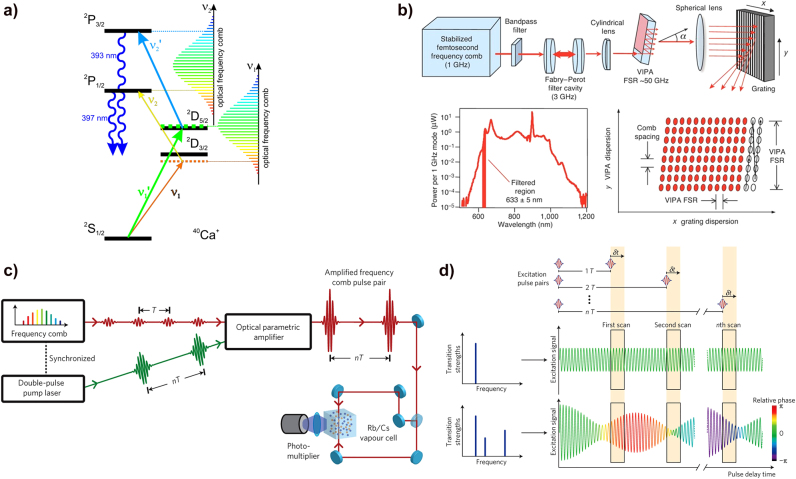
Basic types of optical frequency comb spectroscopy. (a) Direct frequency comb spectroscopy. An optical frequency comb incidents on the targets and some optical modes are absorbed by the excitation of the target atoms or molecules [241]. (b) Dispersive spectroscopy using a pair of VIPA and a grating. The resultant can be expressed as a dot array that depends on both the comb spacing and VIPA FSR [242]. (c) and (d) Ramsey-comb spectroscopy. (c) Experimental setup of spectroscopy. (d) Mechanism of interaction between the target and the pulse pair that have a controllable time difference between each pulse. The excitation signal from the target can be changed based on the time difference of the pulses [243].

There are various representative frequency comb spectroscopic techniques: direct frequency comb spectroscopy [238], Fourier transform spectroscopy [239], and dual-comb spectroscopy [240]. Direct frequency comb spectroscopy is conducted by measuring interactions between light and gas-phase targets by passing them through target samples. The optical frequency comb referenced to an atomic clock provides numerous individual comb lines that are highly precise and stable; for the direct frequency comb spectroscopy, these lines act like a single CW laser, corresponding to each frequency. Thus, these comb lines can contribute to the excitation of a target through a linear or nonlinear phenomenon, while being absorbed as shown in Figure 19a. For a linear process, only a specific, single, or few comb lines, are tuned while interacting with the target and all the other lines remain detuned. In the case of a nonlinear process, many pairs of comb lines with the same sum of frequency contribute to the excitation of the target. Excitation by the optical frequency comb is suitable for spectroscopy through direct absorption and fluorescence detection because it is Doppler-free even when the two pulses are counterpropagating to the target. Thus, direct frequency comb spectroscopy is performed by detecting the comb line absorption or fluorescence of the target using the optical frequency comb for such a target associated optical transition.

In conclusion, this method is useful for simple spectral measurements such as few narrow transitions within the excitation range, and it has the advantage of not requiring a high-resolution spectrometer compared with other spectroscopic techniques. Further, nonlinear frequency conversion can be performed effectively by applying sufficient energy in a short period of time because the optical frequency comb is expressed as a train of ultrashort pulses in the time domain. Further, it is possible to examine and interrogate transitions in the spectral range that cannot be accessed with a CW laser. Thus, this direct optical frequency comb spectroscopy is applied to human exhaled breath, impurities in a semiconductor specialty gas, and a supersonic jet of cold molecules.

Direct frequency comb spectroscopy has been performed through various experimental tools such as dispersive spectroscopy [242] and Ramsey-comb spectroscopy [243]. The basic dispersive spectroscopy is performed through an experimental setup as shown in Figure 19b, and the measurement method is as follows: The comb lines passing through the target are spatially resolved through a pair of virtually imaged phased arrays (VIPA) disperser and a diffraction grating. Then, data are provided in the form of a dots array depending on the spacing of the comb lines and the free spectral range (FSR) of VIPA, each of which can clearly resolve comb lines in the spectral region of interest. Therefore, it is possible to analyze the spectral information of the target by monitoring a spatially resolved dot array because the comb line attenuated by the interaction with the target can be expressed as an intensity variation of the dots.

Ramsey-comb spectroscopy [243] is normally carried out by measuring the interference of atomic or molecular excitations generated through the two amplified pulses by adjusting the time delay, as shown in Figure 19c. For a single pulse interaction, a sinusoidal signal with constant amplitude is generated from the target. However, when two pulse pairs proceed with an integer time delay and sequentially stimulate the target, multi-transitions are excited simultaneously, and therefore, the resultant has a complex amplitude and phase pattern as shown in Figure 19d. The integer multiple of the time delay between the two pulses plays the role of a factor like a repetition rate of an optical frequency comb to generate comb lines with uniform spacing; these comb lines of the time delay allow measuring phenomena such as two-photon excitation. In fact, Ramsey-comb spectroscopy with some specific processes has been reported, and this spectroscopy technique provides an improved frequency accuracy by up to 30 times.

**Figure 20:**
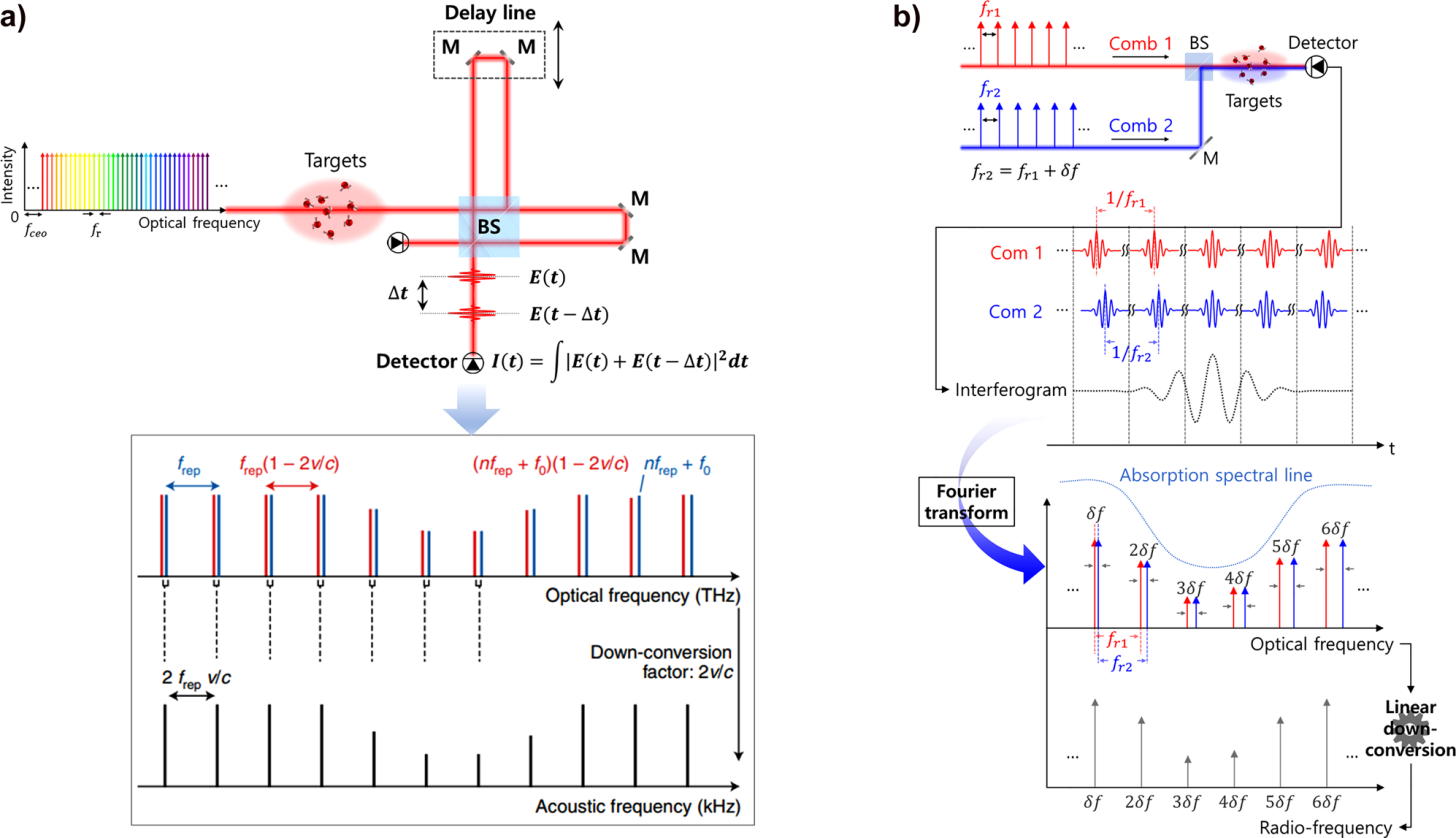
Various frequency comb spectroscopic techniques. (a) Michelson-based Fourier transform spectroscopy using an optical frequency comb. The optical frequency comb has the information of the target by passing through target molecules. The encoded target information can be observed through an interferogram obtained by the two detectors in the form of the equation, $I\left(t\right)=\int {\left|E\left(t\right)+E\left(t-\Delta t\right)\right|}^{2}\text{d}t$ , which is generated by the time delay ∆ t using a delay line. The resultant can be formed in the interval of the Doppler frequency generated by scanning the delay line [112]. (b) Dual-comb spectroscopy. The two optical frequency combs that have a slightly different repetition rate pass the targets. The difference of the repetition rate provides the interferogram using a detector, and it is transformed by the Fourier transform in the frequency domain. The absorption spectral line of the target molecules can be acquired by monitoring the beat frequency between the two combs, which is down-converted to the RF region by a multiheterodyne mixing with a slightly different repetition frequency δf . M, mirror; BS, beam splitter.

Fourier transform spectroscopy with an optical frequency comb is shown in Figure 20a. Here, we plot the measured spectrum, computed by Fourier transforming the coherent optical beat signal between arms 1 and 2 in the time domain. Basic Fourier transform spectroscopy can overcome its limitation of resolution and precision by applying the optical frequency comb as a precise and stable light source [244]. The basic Fourier transform spectroscopy is used as a scanning time-resolved measurement through an optical delay line of a Michelson interferometer; therefore, its resolution greatly depends on the performance of the scanning tools whose resolution is as high as 30 MHz and it is limited by the instrument [111]. Fourier transform spectroscopy with an optical frequency comb is operated through the Doppler effect generated by scanning the delay line. In a Michelson interferometer, the optical frequency combs of both arms interfere with each other by moving the delay line at a constant speed *v*; the resultant is down-converted from the optical frequency to the audio frequency by a factor of $-2\left(f_{\text{ceo}}+nf_{\text{r}}\right)v/c$ because of the small Doppler effect generated by moving the delay line. In addition, this down-converted audio frequency provides the target spectral information, and therefore, the Fourier transform spectroscopy using the optical frequency comb can be self-calibrated by analyzing the audio frequency generated through the interference between the optical frequency comb of each arm; this spectroscopy technique can offer a better resolution regardless of the instrumental limitations because it is operated through a Doppler effect that is not affected by the performance of the scanning tool.

Thus, this Fourier transform optical frequency comb spectroscopy provides better performances in terms of SNR, resolution, and complexity of the spectroscopy. The SNR can be increased by a significantly high brightness achieved by a high coherence and synthesis [242] of the frequency components of the optical frequency comb by generating beats. The mechanism of resolving the optical frequency comb makes it possible to realize a direct self-calibration in the frequency domain and high resolution regardless of the limitation of the instrumental bandwidth; the basic Fourier transform spectroscopy relies greatly on accurate measurements attributed to the positioning of the delay line and a sophisticated approach to systematic effects. Thus, the Fourier transform spectroscopy with the optical frequency comb has been established as an advanced scanning interferometric technique [232, 245]. Furthermore, cavity-enhanced Fourier transform spectroscopy using an optical frequency comb has been actively studied to obtain a better performance. The cavity-enhancement technique is very effective when measuring the spectra of a gas-phase atomic or molecular target with low density. The targets are injected into the cavity installed in the optical path, and the interaction with the targets is enhanced by the light that resonates by the two reflectors of the cavity. The enhanced interaction provides a substantial change to the optical frequency comb, being input the target information to the light multiple times, which increases the SNR, and provides high sensitivity and precision. Owing to these advantages, cavity-enhanced Fourier transform spectroscopy using the optical frequency comb has shown superior results for disentangling the complex spectra of heavy molecules [232, 245].

Various frequency comb spectroscopic techniques have been developed, and thus, a new approach for frequency comb spectroscopy, called dual-comb spectroscopy, is introduced, as shown in Figure 20b 240[240, 246]. Dual-comb spectroscopy, first demonstrated in 2004 [240], provides a direct link between optical and microwave frequencies by down-converting optical bandwidths of tens of terahertz to RF bands on the order of hundreds of megahertz by employing a combination of linear optical sampling with two slightly different repetition rates. Dual-comb spectroscopy has the great advantage of not only obtaining the precision and stability of the comb but also preserving the intensity/phase information of the target samples encoded at the optical frequency comb by direct sampling them from the RF region. Thus, this spectroscopic method allows the molecular absorption spectra to be reconstructed in the RF domain, and thus direct RF analysis. Direct molecular spectrometry using dual-comb spectroscopy provides many benefits compared to conventional methods such as Fourier transform infrared (FTIR) spectroscopy. First, the main advantage is that dual-comb spectroscopy makes it possible to perform measurements with a considerably higher sampling rate than others, regardless of the scanning tool, and to obtain a higher resolution compared to that of scanning spectroscopy, through direct sampling in the RF region by employing two stable optical frequency combs. Dual-comb spectroscopy is independent of the limitations of the instrumental bandwidth and performance because it does not require a complicated optical system.

Recently, dual-comb spectroscopy has been applied in various spectroscopic-based approaches such as photoacoustic dual-comb spectroscopy [247, 248] and coherent anti-stokes Raman spectroscopy (CARS) [234]. Photoacoustic dual-comb spectroscopy is conducted by analyzing photoacoustic signals of the sample using an optical beat-frequency generated by interfering with two different optical frequency combs with slightly different repetition rates. The optical frequency comb is a suitable light source that can provide photoacoustic signals of various wavelengths because it is composed of comb lines that have individual wavelengths in a broadband spectrum. Thus, the photoacoustic dual-comb spectroscopy enables a stable and precise analysis of the photoacoustic signal in the sample without any calibration process, and it makes photoacoustic measurement possible with high resolution, rapid acquisition, and high sensitivity. CARS using dual-comb spectroscopy, first developed by Takuro et al. [234] in 2013, showed superior performance compared to other techniques. This spectroscopy method measures anti-stoke Raman scattering with two-photon Raman excitation using two optical frequency combs with slightly different repetition rates. The two optical frequency combs are represented as pulses with a period corresponding to the reciprocal of the repetition rate on the time domain; anti-stoke Raman scattering is measured based on various factors generated using the time delay between the pulses reaching the target. CARS using dual-comb spectroscopy, which was set with a repetition rate difference of 5 Hz, could precisely resolve each substance in a mixture of liquid chemicals with a resolution of about 4 cm^−1^. Furthermore, it shows great improvement in the spectral span, SNR, measurement time, and several spectral elements. In conclusion, the dual-comb technique could greatly overcome the limitations of the existing ones and contribute to the progress of spectroscopy.

However, despite the many advantages of frequency comb spectroscopy, there are some inevitable difficulties: the most fundamental challenge with frequency comb spectroscopy is that the intensity of each comb line is too low, and it essentially requires an optical cavity [249], [250], [251] with a pair of reflectors, which becomes systematically complex and easily affected by external noises when a sufficient SNR for the measurement is required. Spectral magnitude fluctuations attributed to external noises set up a challenge, especially when interrogating broad molecular transitions or randomly clustered molecular spectra. Further, in the case of dual-comb spectroscopy, a sophisticated controlling process must be involved to conduct the measurements, such as the accurate controlling of the slight differences between the two optical frequency combs.

### 3.3 Frequency-comb-referenced plasmonic phase spectroscopy

We provide details of the ultraprecision spectroscopy for one application in ultrafast plasmonics – frequency-comb-referenced (FCR) plasmonic phase spectroscopy – by combining an optical frequency comb and a plasmonic nanostructure.

The monitoring of plasmonic gap with both ultrahigh resolution and precision is crucial to observe quantum mechanical effects such as electron tunneling and nonlocal screening wherein the plasmonic active local area should be controlled at the sub-nanometer scale [252]. Despite the frequent mention about observing the phenomena at a sub-nanometer scale, such ultraprecision ruler for measuring the sub-nanometer scale length or gap change in the nanostructures is yet to be explored. The direct measurement of a broadband phase spectrum [239, 253] may allow overcoming the limitations of previous spectroscopic techniques in terms of resolution and precision by providing linear, direct, and abundant information of targets compared with spectral intensity [9, 254], commonly used by most plasmonic spectroscopic techniques. The intensity-based measurement has a fundamental limitation in that a precise quantitative comparison is difficult because of the intensity fluctuations and low-frequency noises generated by the environment; the importance of phase spectrum analysis is growing gradually because of its high precision, needed to achieve better spectroscopic performance. Therefore, there have been attempts to develop a broadband phase spectroscopy [255], [256], [257], but these were approaches to measure indirect phase information based on the intensity spectrum and were affected by low-frequency noises. Thus, there is a limit to the appearance of sub-nanometer plasmonic spectroscopy.

The development of the FCR plasmonic phase spectroscopy facilitates the activation and realization of sub-nanometer length measurements. First, the optical frequency comb makes it possible to overcome prior limitations of spectroscopy such as resolution, precision, and effectiveness of low-frequency noises by applying its ultrahigh phase coherence, stable, and broad spectral bandwidth. The LSPR generated by the plasmonic sample implemented in this spectroscopic technique makes it very sensitive to changes in the geometric parameters of the sample, and in the local refractive index induced by biomolecular binding and molecular composition changes. Further, in terms of atomic or molecular spectroscopy, there is one more great advantage; the enhanced near-field obtained through the plasmonic effect generates a light-induced force in a local area. This optical force can trap a gas-phase target, even a single atom in a localized space, and this effect of optical tweezing provides high sensitivity, which allows the detection of a low-density target. According to all these advantages, FCR plasmonic phase spectroscopy can obtain high sensitivity based on the various phenomena attributed to the plasmonic effect, and the ultrahigh-resolution and precision achieved using the optical frequency comb.

**Figure 21:**
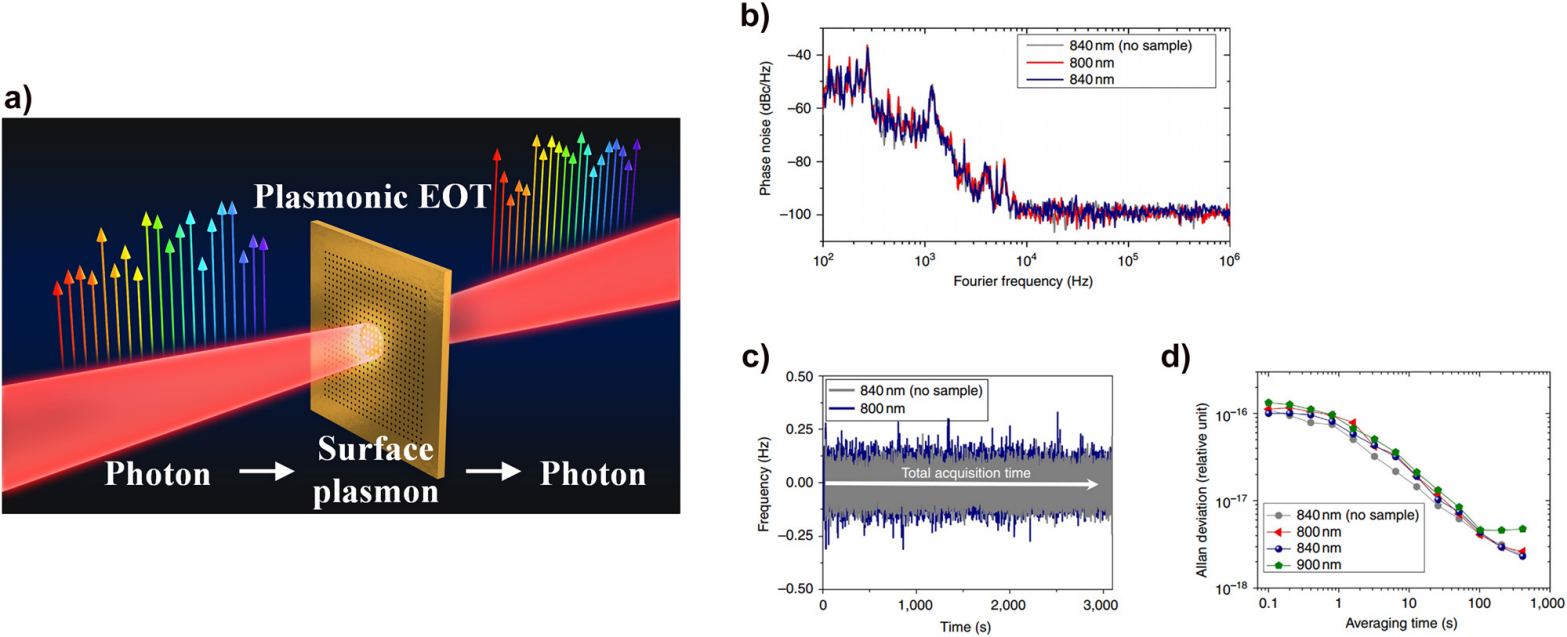
Plasmonic conversion of the optical frequency comb. (a) The frequency comb experiences photon-SP-photon conversion by passing through a plasmonic nanostructure. (b)–(d) analysis of the plasmonic frequency comb: phase noise and frequency stability. (b) Phase noise spectra at a 1.2 GHz RF carrier at three different cases; on-resonance wavelength of 840 nm, off-resonance wavelength of 800 nm, and without plasmonic EOT at the wavelength of 840 nm. (c) Time trace of the RF beats with and without the plasmonic EOT over 3000 s. (d) Allan deviations of frequency stability with changing the average time for different wavelength positions at on- and off-resonance conditions [258].

It is necessary to examine the optical frequency comb in terms of its frequency components and linewidth, which can be degraded by the interaction between the comb and SPs by passing through a plasmonic sample, to implement FCR plasmonic phase spectroscopy by fusing the optical frequency comb and plasmonic sample; this process was demonstrated experimentally by Xiao et al. [258] using a metallic nanohole array used for plasmonic EOT samples, as shown in Figure 21a. In the experiment, the frequency components and linewidths of the plasmonic comb were accurately investigated through the RF beats generated by the interference between two optical frequency combs using a heterodyne Mach–Zehnder interferometer. The reference frequency comb without passing the EOT samples was frequency-shifted by an AOM, resulting in an optical RF beat frequency between the reference and measured frequency combs, equivalent to the AOM frequency if the plasmonic transformation does not affect the characteristics of the optical frequency comb. The measurement frequency comb experiences photon-SP-photon conversion by passing through the plasmonic EOT sample located at the measurement beam path. The frequency components influenced by the plasmonic conversion, which can be encoded in the far-field because of the high coherence of the comb and SPs, were inspected by analyzing the RF beat frequency. Thus, no degradation of the optical frequency comb was observed by monitoring the RF beats with various RF spectra and analyzing them precisely using a 1 Hz video bandwidth (instrumental limitation). Both phase noise and frequency stability were well-preserved, as shown in Figure 21b–d; further, specific phase noises were not produced by the plasmonic samples, and this was verified by checking the long-time phase noise measurement. Finally, following all these demonstrations, it was possible to apply an optical frequency comb as a light source to plasmonic phase spectroscopy while maintaining its superior performance.

**Figure 22:**
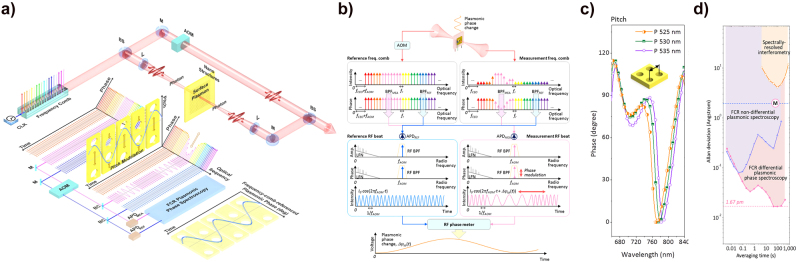
FCR plasmonic phase spectroscopy. (a) Principle of the FCR differential plasmonic phase spectroscopy. The frequency comb at the measurement beam path passing through the plasmonic structure suffers a plasmonic phase change and interferes with the reference frequency comb, which frequency is shifted by the AOM. The beat resultants by coupling the two combs are down-converted to the RF domain, wherein the state-of-the-art RF electronics and time standards can be applied. The output RF electric field oscillations from the plasmonic on and off-resonance position are detected by a pair of photodetectors. Their relative phase difference is measured precisely with a phase meter referenced to a time standard. When a small dynamic motion is applied to the plasmonic sample as a subnanometer thermodynamic pitch modulation, the FCR plasmonic phase spectroscopy can measure it with an ultrahigh-resolution at a high update rate. (b) Spectral phase changes of the different hole pitches; there is a steep phase peak around the resonance wavelength. (c) Spectral noise suppression and differential phase detection; the optical frequency of the reference comb is upshifted by an RF frequency of $f_{\text{AOM}}$ , which works as the information carrier in the subsequent RF signal band-pass-filtering, amplification, and phase detection. This can enable the effective suppression of all noise located other than $f_{\text{AOM}}$ . A differential phase detection scheme is implemented to further suppress the low-frequency noise. An RF phase meter measures the final phase difference referencing it to a time standard. (d) The analysis of the resolution by Allan deviation. Compared to the comb-based spectrally resolved interferometry, FCR differential plasmonic phase spectroscopy provides stability and faster response time. The latter is improved by three orders of magnitude. CLK, atomic clock; M, mirror; L, lens; AOM, acousto-optic modulator; BC, beam combiner; APD, avalanche photodiode; BPF, bandpass filter; LFN, low-frequency noise [14].

The FCR plasmonic phase spectroscopy was first developed by Ahn et al. as shown in Figure 22a in 2018 [14]. The key points of this spectroscopic technique are the optical frequency comb, SPR, plasmonic phase spectrum, and RF signal processing. First, the optical frequency comb referenced to an atomic clock allowed a linear extraction of the direct phase spectrum. In the experiment, the optical frequency comb was phase-locked to a reference rubidium atomic clock and has a broad spectral bandwidth, from 530–950 nm, with a central wavelength of 780 nm and a repetition rate of 250 MHz. The stabilized frequency comb provides ultrahigh precision in resolving the phase spectrum stably through millions of well-defined and phase-coherent comb lines.

Second, the LSPR generated by the interaction between frequency combs and electrons in the plasmonic EOT was extremely sensitive to structural transitions of the plasmonic nanostructures while preserving the superior optical properties of the comb. Furthermore, the plasmonic resonance effect played a role as the optical resonator like a Fabry–Perot cavity, which amplified the interaction between the SPs and nanostructures.

Third, Anh et al. demonstrated that a narrow phase peak with a steep slope was generated around the resonance wavelength of the plasmonic EOT as shown in Figure 22b; this served as a useful indicator to perform broadband phase spectroscopy. For many spectroscopic techniques, it is necessary to identify specific spectral peaks that can be a reference factor for sensing based on spectral peak shift measurements; therefore, the phase spectral peak around the resonance wavelength allowed the realization of an ultraprecision phase sensing of the plasmonic dynamic motion induced by an isotropic thermal expansion by monitoring the direct phase spectrum. For an accurate analysis, the contribution of each geometric parameter, such as the hole pitch, diameter, and thickness of plasmonic EOT as the phase changes, was calculated by numerical simulations. It was numerically confirmed that the phase change was dominated by the pitch of the metallic nanohole array (approximately 97.1%); this implies that the picometre target detected by the FCR plasmonic phase spectroscopy was the plasmonic dynamics induced by the thermodynamic motion of the hole pitch of the plasmonic EOT.

The last key was the RF signal processing shown in Figure 22c, which means that the optical signal obtained through the FCR plasmonic phase spectroscopy was analyzed using a differential RF signal process using RF electronics referenced to an atomic clock:

(1) Fundamental optical RF beat signals were obtained using a heterodyne Mach–Zehnder interferometer. The low-frequency noise can be removed by a frequency shift with an AOM and spectral filtering. The phase retardation value induced by a plasmonic resonance of the target is encoded in the RF beat signal.
(2) The RF beat signals observed by a photodetector are additionally super-down-converted to a lower frequency regime. This procedure is needed to extract the phase value with RF electronics. The instrumental phase measurement resolution is limited by the bandwidth of the RF electronics; therefore, confirming the benefits of the measured frequency down-conversion.
(3) Most optical systems suffer environmental noise problems, such as airflow, temperature, and humidity changes that hinder the precise measurement of phase changes from the target sample. When broadband optical frequency combs illuminate the plasmonic target, the plasmonic phase retardation occurs only at around the resonance wavelength. However, frequency combs at both resonance and nonresonance wavelength suffer phase variations by the same environmental noise that could be subtracted in the phase measurement value, as shown in Figure 22a. Therefore, the noises in the RF beats of the two wavelengths were canceled out, and they retain then only the pure phase information induced by the plasmonic dynamics motion.

In conclusion, the FCR plasmonic phase spectroscopy was implemented by combining all key points of plasmonics and optical frequency comb, which was demonstrated experimentally, as shown in Figure 22a; this spectroscopic technique is able to capture a plasmonic dynamic motion of 1.94 Å with a 1.67 pm resolution at a 200 Hz sampling rate, as indicated in Figure 22d. The performance of the FCR plasmonic phase spectroscopy shows not only a resolution that was two orders of magnitude better than prior high-resolution spectroscopy [259] but also three orders of magnitude faster measurement speed. This unprecedented state-of-the-art spectroscopic technique opened up the possibility of the real-time monitoring of the sub-nanometer dynamical processes; this was difficult to assess before, and now, it has the potential for application to several scientific fields including physics, chemistry, and biology.

## 4 Summary and perspectives

Even though the ultrafast plasmonics in strong-field physics has opened up new possibilities in manipulating the electrons’ dynamics with a highly enhanced local field, the thermal damage threshold in metallic nanostructures hinders the competitive performance when compared with conventional CPA-based laser experiments. Similarly, reliable, large-scale, and cost-effective fabrication techniques should be investigated intensively for broad uses of SP-based precision spectroscopy.

The use of different materials for plasmonics, not gold, could be the way to resolve the aforementioned issues. Modern optical technologies allow a wide spectral range of ultrafast pulses from the visible to the infrared, inducing plasmonic effects in nonmetallic materials, such as silicon or graphene.

We hope to deliver to the readers a concise and deep understanding of SPs and associated ultrafast applications and provide helpful guidelines for investigating these novel ultrafast plasmonic phenomena and devices through these brief discussions.
